# SGRL can regulate chlorophyll metabolism and contributes to normal plant growth and development in *Pisum sativum* L.

**DOI:** 10.1007/s11103-015-0372-4

**Published:** 2015-09-07

**Authors:** Andrew Bell, Carol Moreau, Catherine Chinoy, Rebecca Spanner, Marion Dalmais, Christine Le Signor, Abdel Bendahmane, Markus Klenell, Claire Domoney

**Affiliations:** John Innes Centre, Norwich Research Park, Norwich, NR4 7UH UK; INRA/CNRS – URGV, 2 rue Gaston Crémieux, 91057 Evry, France; INRA, UMR 1347 Agroécologie, Dijon, France; School of Biological Sciences, University of Essex, Wivenhoe Park, Colchester CO4 3SQ UK

**Keywords:** Chlorophyll turnover, Pea, SGR, Staygreen, TILLING mutants, Transient expression

## Abstract

Among a set of genes in pea (*Pisum sativum* L.) that were induced under drought-stress growth conditions, one encoded a protein with significant similarity to a regulator of chlorophyll catabolism, *SGR*. This gene, *SGRL*, is distinct from *SGR* in genomic location, encoded carboxy-terminal motif, and expression through plant and seed development. Divergence of the two encoded proteins is associated with a loss of similarity in intron/exon gene structure. Transient expression of SGRL in leaves of *Nicotianabenthamiana* promoted the degradation of chlorophyll, in a manner that was distinct from that shown by SGR. Removal of a predicted transmembrane domain from SGRL reduced its activity in transient expression assays, although variants with and without this domain reduced SGR-induced chlorophyll degradation, indicating that the effects of the two proteins are not additive. The combined data suggest that the function of SGRL during growth and development is in chlorophyll re-cycling, and its mode of action is distinct from that of SGR. Studies of pea *sgrL* mutants revealed that plants had significantly lower stature and yield, a likely consequence of reduced photosynthetic efficiencies in mutant compared with control plants under conditions of high light intensity.

**Electronic supplementary material:**

The online version of this article (doi:10.1007/s11103-015-0372-4) contains supplementary material, which is available to authorized users.

## Introduction

Colour is an important quality trait in many plant foods and organs, including leaf vegetables and seeds, and is influenced by a combination of factors, including the relative abundance of chlorophyll and carotenoids. The trait is also linked with plant health and can reflect disease and stress symptoms, senescence, or a combination of both. The vegetable industry seeks the uniform retention of strong green colour in certain products, to meet the demands of lucrative food markets. Products that fail to meet the high quality standards of visual appearance for human consumption markets or have limited shelf-life will offer a much lower return to producers. Historically, such quality traits have been improved by breeding and selection, without knowledge of the effects of individual genes involved in their control. In pea, mutants that retain chlorophyll in their cotyledons during and following senescence have been exploited for vegetable markets, for both fresh and dried seed products. It is only in recent years that the biochemical steps involved in chlorophyll breakdown have been elucidated in detail for a number of crops, and in *Arabidopsis thaliana* (L.) Heynh. (Chung et al. 2006; Ren et al. 2007; Aubry et al. 2008; Horie et al. 2009; Morita et al. 2009; Schelbert et al. 2009; Buchert et al. 2011; Hörtensteiner and Kräutler 2011; Christ and Hörtensteiner 2014; Sakuraba et al. 2013).

The chlorophyll degradation pathway is initiated usually during senescence in higher plants and converts chlorophyll to colourless breakdown products that accumulate in the vacuoles, causing yellowing of leaves and cotyledons due to carotenoid exposure (Hinder et al. 1996; Hӧrtensteiner 2006). The multi-step conversion pathway eliminates potential phototoxic chlorophyll catabolites and permits the remobilisation of nitrogen from chlorophyll-binding proteins (Hӧrtensteiner and Feller 2002; Hӧrtensteiner and Kräutler 2011; Thomas and Ougham 2014). The failure to degrade chlorophyll can reflect a lesion in one of several components of the degradation pathway, leading to functional or non-functional (cosmetic) stay-green phenotypes. The latter phenotype is usually the consequence of a so-called type C stay-green mutation, which allows other aspects of senescence to proceed as normal but with impaired chlorophyll degradation (Thomas and Smart 1993; Thomas and Ougham 2014).

Recent work has suggested a generalised model for chlorophyll breakdown, which may be applicable to senescence processes in most plant organs. In this model, chlorophyll *b* is converted to chlorophyll *a* by a two-step reduction involving three proteins. Two of these (NOL, NYC1) act as a chlorophyll *b* reductase (CBR) to reduce chlorophyll *b* to 7-hydroxy-methyl chlorophyll (HMC) *a*, which is further reduced by HMC *a* reductase to chlorophyll *a* (Horie et al. 2009; Kasuba et al. 2007; Meguro et al. 2011). CBR has also been suggested to act as a key component of Light Harvesting Complex II (LHCII) degradation (Horie et al. 2009; Kasuba et al. 2007). In a non-senescing plant the ratio of chlorophyll *a*/*b* is regulated by CBR with the reverse reaction being catalysed by chlorophyllide *a* oxygenase (Tanaka et al. 1998; Espineda et al. 1999; Scheumann et al. 1996).

During chlorophyll degradation, removal of the central magnesium (Mg) ion and the later removal of the hydrophobic phytol side chain by pheophytin pheophorbide hydrolase (PPH) have been shown to precede the porphyrin ring opening step, which is catalysed by pheophorbide *a* oxygenase (PaO) (Schelbert et al. 2009; Hörtensteiner and Kräutler 2011). The product of PPH is pheophorbide *a*, the final green pigment in the pathway (Schelbert et al. 2009), which is oxygenolytically converted by PaO to the transient intermediate, red chlorophyll catabolite (RCC). This last metabolite is metabolically channelled via a proposed interaction between PaO and RCC reductase to form primary fluorescent chlorophyll catabolites (Hӧrtensteiner et al. 1998; Pružinská et al. 2007), which are exported from the plastid and undergo extensive modifications in the cytosol and, following import into the vacuole, accumulate as non-fluorescent chlorophyll catabolites (Oberhuber et al. 2003; Hinder et al. 1996; Matile et al. 1992).

Recent research in several crop and model plants has shown that the chlorophyll degradation pathway is controlled overall by a regulatory protein, SGR, which is maximally expressed during senescence. However, the precise mechanism by which SGR functions remains unclear and, despite the high levels of homology between species, no precise catalytic domain has been defined experimentally (Hörtensteiner and Kräutler 2011; Park et al. 2007). Yeast hybrid assays and in vivo and in vitro experiments have indicated interactions between most of the enzymes believed to be involved in the chlorophyll degradation pathway, including SGR. It has been suggested that SGR may play a role in the recruitment of these enzymes to LHCII (Sakuraba et al. 2012). Interestingly, a point mutation in a rice *sgr* mutant showing a stay-green phenotype still displayed the accumulation and binding of SGR at LHCII, suggesting that SGR could have an additional unknown enzymatic function as well as a recruitment role (Hörtensteiner and Kräutler 2011; Park et al. 2007). Stay-green phenotypes have been described for mutants of *SGR* in a variety of species, including pea, where genetic variation in *SGR* was shown to co-segregate with the *i* locus, determining cotyledon colour (Armstead et al. 2007; Aubry et al. 2008).

In this paper, we describe the identification and characterisation of a SGR-like protein in pea, which shows several distinctive features that contrast with those of SGR. This protein, SGRL, is capable of metabolising chlorophyll in a manner that differs from SGR. Investigations of the activities of both proteins, using transient expression in *Nicotiana benthamiana* and biochemical analysis of phenotypes obtained by co-expression assays, suggested that the two proteins have roles in distinct developmental processes. Assays of wild-type and mutant derivatives of SGRL have suggested protein regions associated with activity. Mutant pea plants generated by TILLING and encoding a truncated SGRL showed impaired growth, and reduced photosynthetic efficiency under high light intensity, in support of a proposed role for SGRL during normal developmental processes in plants.

## Materials and methods

### Plant materials and growth conditions

Seeds from the JIC *Pisum* germplasm collection and TILLING lines (http://www-urgv.versailles.inra.fr/tilling/index.htm; Dalmais et al. 2008) were grown in JIC glasshouses for plant material and trait analysis, with supplementary heat and light in winter months. A set of diverse lines, comprising *Pisum* germplasm accessions and cultivars (19 lines: JI 2822, JI 185, JI 73, JI 1294, JI 813, JI 2775, JI 281, JI 399, JI 3129, JI 1201, JI 1194, JI 2202, JI 15, *cv*. Cameor, *cv*. Brutus, *cv*. Birte, *cv*. Kahuna, *cv*. Princess and *cv*. Enigma), was used to screen for genetic diversity. *Nicotiana benthamiana* plants for transient expression assays were grown in a JIC containment glasshouse for 5–6 weeks before use. TILLING lines were grown to select homozygous mutant genotypes and multiplied to the M5 generation to provide enough seeds for comparative purposes. For comparisons of the pea *sgrL*^*3421*^ (W197^STOP^) mutant and control (*cv*. Cameor) plants, two independent experiments were performed using either 20 (plant trait measurements) or 50 (plant trait and biochemical measurements) replicate plants of each line. Plants were grown in 9 cm^2^ pots and watered normally for 27 days. Then half of each group was maintained under either well-watered or drought-stress conditions, following procedures previously described (Charlton et al. 2008), with modifications for the latter treatment. At the onset of the drought treatment, plants were not watered for nine days and, thereafter, these plants were given 20 ml of water per day throughout the recovery period until plants senesced. Biochemical data were collected on the final day on which water was withheld and 7 and 13 days later (Recovery Day (RD) 0, RD7 and RD13). Climate data for RD0, 7 and 13 were collected on the John Innes Centre site using a T200 Horticultural Computer (a TomTech weather station).

### Library construction and sequence identification

RNA preparations from well-watered or drought-stressed plants (all as described in Charlton et al. 2008) were used for the construction of libraries and suppression subtractive hybridisation (SSH) carried out to identify those transcripts that were drought-responsive. Individual RNA batches were tested for induction of dehydrin, as a control drought-stress responsive sequence (Charlton et al. 2008), where expression of a His-Asp phosphorelay gene (GenBank AJ831475.1) was used as a control (Supplementary Fig. S1). A pea SSH cDNA library, enriched for drought-responsive leaf cDNAs, was constructed (Clontech PCR-Select™ cDNA Subtraction Kit) with checks throughout on the quality of the polyA+ RNA, cDNA synthesis, ligation of the adapter primers and the subtractive hybridisations. Subtracted cDNA was cloned into the plasmid vector pCR2.1 (Invitrogen) and transformed into a super-competent *E. coli* strain (One Shot TOP 10, Invitrogen). Sequence analysis of clones identified 557 unigenes (database accessions EBI AM161647-AM162203), and these were classified into groups according to likely function (Supplementary Fig. S1). Representative clones were chosen to examine the ratio of transcript level in drought-stressed compared with well-watered plants across four independent experiments. Amplified PCR products from 96 clones (1 µl from 10 µl purified product spotted on duplicate nylon filters) were hybridized with digoxygenin-labelled total cDNA from control and stressed plants. Quantification of the relative signal from images of the filters provided a conservative estimate that over 15 % of the products were up-regulated in the drought-stressed cDNA.

### Genomic analysis

DNA was prepared from pea leaves using a manual extraction method (Welham and Domoney 2000). Genomic DNA amplification was performed using TaKaRa Ex Taq (Clontech-Takara Bio Europe), according to the manufacturers’ instructions, with the following PCR conditions: 98 °C for 10 s, 58 °C for 30 s, 72 °C for 1 min per kb amplified fragment, final extension at 72 °C for 5 min, hold at 4 °C. Sequencing of genomic amplicons was performed by TGAC (tgac.ac.uk) and Eurofins (eurofinsgenomics.eu) sequencing services. The primers used for genomic and cDNA amplification and sequencing are available in Supplementary Table 1.

### Genetic mapping of SGRL

Mapping in the JI 281 × JI 399 population was carried out using 91 progeny lines and exploiting a Cleaved Amplified Polymorphic Sequence (CAPS) marker, based on the enzyme *Ssp*I to digest an amplicon, generated by the primers MK_C15F and MK_C15rev (Supplementary Table 1). Recombination frequencies and marker associations were estimated using THREaD Mapper (Cheema et al. 2010) and Haldane functions (Haldane 1919). Mapping in the Princess × JI 185 population was carried out using 152 progeny lines and performed by sequence analysis of a single nucleotide polymorphism (SNP) in an amplicon generated by the primers, SGRL-F8 and SGRL-R1, and sequenced using the primer SGRL-R3 (Supplementary Table 1); data were analysed using JoinMap (Stam 1993).

### cDNA and qRT-PCR analysis

Total RNA was isolated from tissues which were frozen in liquid nitrogen, and stored at −80 °C. Tissues were either powdered directly or after freeze-drying (for high water content tissues) and ground in 700 µl RNA extraction buffer (1 M Tris–HCl pH 9.0, 1 % SDS, 10 mM EDTA), extracted twice with 350 µl phenol and 350 µl chloroform/IAA (24:1) and centrifuged at 14,000 rpm for 5 min. Following addition of 50 μl 3 M sodium acetate and 1 ml 100 % ethanol to 500 μl of supernatant, RNA was precipitated at −80 °C for 1 h, recovered by centrifugation at 14,000 rpm for 5 min, dried and dissolved in 200 µl RNAse-free water, and precipitated by addition of 200 µl 4 M LiCl overnight at 4 °C. RNA pellets were washed with 900 µl 2 M LiCl, twice with 900 µl 100 % ethanol, centrifuged, dried and dissolved in 25 µl RNase-free water.

RNA samples were DNase-treated (Qiagen RNeasy Mini Kit) prior to first strand cDNA synthesis, which was carried out using 1–3 µg RNA and 10 pmol primer A236 (poly A adaptor) in 11 µl H_2_O which was heated to 70 °C for 10 min, immediately cooled on ice and centrifuged briefly. Following addition of 4 µl enzyme reaction buffer, 2 µl 0.1 M DTT, 1 µl RNase inhibitor cocktail (Invitrogen), 1 µl dNTP (10 mM) and 1 µl Superscript II reverse transcriptase (Invitrogen), reactions were incubated at 37 °C for 2 h. To obtain the 5′ untranslated SGRL sequence, a 5′ RACE system (Invitrogen) was used, according to the manufacturer’s instructions, with A236 primer as GSP1, and using SGRL R4 and R7 primers (Supplementary Table 1) in the nested PCR step with the AAP and AUAP kit primers, respectively.

Quantitative real-time PCR (qPCR) amplification of first strand cDNA templates from a variety of plant organs and the subsequent quantification of SGR and SGRL products was carried out, essentially under conditions described (Hellens et al. 2010; Chinoy et al. 2011), and using pea actin as a reference gene (Cooper et al. 2005). In each analysis the data were calibrated against the lowest expressing tissue (assigned a value of 1). The specificity of the primers was confirmed by gel analysis of products and melting curve analysis, and the authenticity of the products was verified by sequencing. The mean relative gene expression levels presented were determined from three independent experiments. Analysis of RNA from developing seeds at 10, 20, 30 days after flowering provided contrasting developmental stages (Vigeolas et al. 2008). For comparisons of gene expression during leaf development, leaves were sampled according to phenotype. Very young leaves were close to the growing apex within 1–2 days of unfurling. Young to mature leaves were fully expanded leaves near the apex or mid-way along the length of fully-grown plants. Old and very old leaves were those showing some loss of colour (wild-type *SGR*) and/or structure (mutant *sgr*).

### Suppression PCR and gene walking

Using a method adapted from that of Siebert et al. (1995), 15 restriction digests of pea genomic DNA (*Bam*HI, *Bcl*I, *Bgl*II, *Bst*YI, *Cla*I, *Msp*I, *Taq*I, *Dra*I, *Hpa*I, *Eco*RV, *Nae*I, *Sca*I, *Pvu*II, *Ssp*I and *Stu*I) were performed (5 μl DNA, 1 μl restriction enzyme, 8 μl 5× RL buffer (50 mM Tris acetate pH 7.5, 50 mM Mg acetate, 250 mM K acetate, 25 mM dithiothreitol, 250 ng/μl bovine serum albumin, 26 μl H_2_O)). Digests were incubated overnight at the appropriate temperature for each restriction enzyme. The digests were ligated by adding 2 μl 5× RL buffer, 1 μl T4 ligase (Gibco BRL Life Technologies), 5 μl 10 mM ATP, 1 μl adaptor primer appropriate to the enzyme (Supplementary Table 1) and 1 μl H_2_O, and incubation for 6 h at room temperature. Ligated restriction digests were diluted 1:1 with T0.1E (10 mM Tris–HCl, 0.1 mM EDTA) pH 8.0 before PCR amplification. Nested PCR was based on two gene-specific primers, the adaptor primers APX1A and APX1B (Supplementary Table 1) and the standard PCR master mix. The PCR conditions were: 94 °C for 2 min, 20 cycles of: 94 °C for 1 min, 65 °C for 30 s and 72 °C for 2 min, followed by a final 5 min extension at 72 °C and held at 4 °C for the first PCR; the second PCR conditions were: 94 °C for 2 min, 5 cycles of: 94 °C for 1 min, 65 °C for 30 s and 72 °C for 2 min, followed by 40 cycles of: 94 °C for 1 min, 62 °C for 1 min and 72 °C for 2 min, a final 5 min extension at 72 °C and held at 4 °C.

### GATEWAY BP and LR cloning

Infiltration of leaves of *Nicotiana benthamiana* (adapted from Sainsbury et al. 2009) was exploited as a system to transiently express genes of interest and monitor phenotype. Coding sequences were amplified from first strand cDNA samples, using attB adaptor primers (Supplementary Table 1) in high fidelity PCR (Phusion polymerase, New England BioLabs), according to the manufacturer’s instructions, and the following conditions: 98 °C for 1 min, 5 cycles of: 98 °C for 10 s, 56 °C for 30 s, 72 °C for 1 min per 1 kb to be amplified, followed by 30 cycles of: 98 °C for 10 s and 72 °C for 1 min per 1 kb to be amplified, and a final elongation at 72 °C for twice the elongation time in the previous cycles, before being held at 4 °C. PCR products were cleaned using Wizard^®^ SV Gel and PCR Clean-up System (Promega), according to the manufacturer’s instructions, before cloning. Coding sequences were cloned by GATEWAY via two reactions into pEAQ-HT Dest vectors (GenBank GQ497237.1) and subsequent transformation into *Agrobacterium*.

For the BP reaction, 1 µl of BP Clonase (Invitrogen BP Clonase kit) was added to 1 µl of Phusion PCR product, 1 µl pDONR207 (Invitrogen) and 2 µl TE buffer and left at 25 °C overnight. The enzyme was inactivated by adding 0.5 µl Proteinase K with incubation at 37 °C for 10 min. An aliquot (1 µl) of BP (or LR) reaction was added to 50 μl competent DH5α *E. coli* (Invitrogen) or 25 µl One Shot^®^ TOP10 Chemically Competent *E. coli* (Invitrogen) cells and left on ice for 30 min. Cells were heat-shocked at 42 °C for 45 s and immediately cooled on ice. Recovery was in 900 or 450 µl S.O.C. medium (2 % tryptone, 0.5 % yeast extract, 10 mM NaCl, 2.5 mM KCl, 10 mM MgCl_2_, 10 mM MgSO_4_, 20 mM glucose) for the different cells, respectively (37 °C, shaking for 2 h). Aliquots of 20 and 50 µl were spread onto LB agar plates (tryptone 10 g L^−1^, yeast extract 5 g L^−1^, NaCl 10 g L^−1^, 1.1 % agar (pH 7.0)), supplemented with the appropriate antibiotic (gentamicin for BP cultures; kanamycin for LR cultures) and grown overnight at 37 °C.

PCR was performed on individual colonies, which were sampled first into PCR mix and then into 50 µl LB (as above without agar), supplemented with the appropriate antibiotic(s) (gentamicin for BP colonies; kanamycin for LR colonies; rifampicin, kanamycin and tetracycline for *Agrobacterium* colonies (see later)). Colony PCR conditions were 94 °C for 2 min, 35 cycles of: 94 °C for 15 s, 56 °C for 30 s, 70 °C for 1 min per 1 kb DNA amplified, followed by 4 °C hold. PCR amplicons were verified by sequencing and validated clones grown overnight at 37 °C with shaking (28 °C for *Agrobacterium*). Plasmids were extracted using the Wizard^®^ PLUS SV Minipreps DNA purification system (Promega), according to the manufacturer’s instructions.

For the LR reactions, 1 µl of LR Clonase (Invitrogen LR Clonase kit) was added to 1 µl of BP plasmid DNA, 1 µl of pEAQ-HT DEST 1 or DEST 3 (pEAQ-HT vector and modifications for GATEWAY compatibility, Sainsbury et al. 2009) and 2 µl TE buffer, and incubated at 25 °C overnight. The enzyme was inactivated by adding 0.5 µl Proteinase K (Invitrogen) and incubation at 37 °C for 10 min, and plasmids cloned and selected as described above.

### *Agrobacterium* transformation and agro-infiltration

A 2 µl aliquot of LR plasmid DNA was added to 50 µl electro-competent *Agrobacterium* cells (C58C1) and electroporation performed at 2.5 V for 4.5–4.8 s. Cells were recovered in 1 ml LB broth with shaking at 28 °C for 2 h. Aliquots (50 and 100 µl) were plated onto LB agar supplemented with rifampicin, kanamycin and tetracycline and grown at 28 °C for 2 days before colony selection as above.

*Agrobacterium* cells were recovered by centrifugation, re-suspended in MMA solution (1 ml 1 M MES, 1 ml 1 M MgCl_2_, 150 µl 0.1 M acetosyringone, in 100 ml H_2_O), diluted to OD_600_ 0.4 ± 0.025 (unless otherwise stated) and shaken at room temperature for 3 h. Cells were infiltrated into leaves of *Nicotiana benthamiana* plants (5–6 weeks old) by piercing the back of the leaf with a needle and injecting the re-suspended cells into the pierced hole using a syringe. Up to four samples were infiltrated into a single leaf, with between six and nine replicates for every construct tested per experiment. For double infiltration experiments, a gene encoding Green Fluorescent Protein (GFP) was used as a control that was not related directly to the chlorophyll metabolic pathway. For dark incubation experiments, leaves were covered with a cardboard box and an outer black bag immediately following infiltration.

### Evaluation of leaf phenotypes

A Photosynthesis Efficiency Analyser (PEA) (Hansatech, UK) was used to analyse photosynthetic activity of leaves, using a number of parameters provided by the analyser; Fv/Fm gave a measure of photosystem II efficiency. Chlorophyll assays were carried out, using discs of 5 mm diameter from leaf samples, which were freeze-dried, ground with acid-washed sand and extracted in 500 μl of 10 mM Tris–HCl buffered, pH 8.0, 80 % acetone for 30 min at 0 °C (*Nicotiana benthamiana*) or in 100 % acetone overnight at −20 °C (pea). Samples were centrifuged for 2 min at 14,000 rpm and absorbance at 664 and 647 nm measured, using 450 μl of supernatant. Chlorophyll concentrations were determined (Porra 2002), where:

$${\text{Chl}}a\left( {\upmu{\text{g}}/{\text{ml}}} \right) = \left( {12.25 \times A664} \right){-}\left( {2.55 \times A647} \right)$$

$${\text{Chl}}b\left( {\upmu{\text{g}}/{\text{ml}}} \right) = \left( {20.31 \times A647} \right){-}\left( {4.91 \times A664} \right)$$

$${\text{Chl}}a + b\left( {\upmu{\text{g}}/{\text{ml}}} \right) = \left( {17.76 \times A647} \right) + \left( {7.34 \times A664} \right)$$

### Evaluation of transient gene expression using His-tagged proteins

All the constructs assembled for transient expression in *Nicotiana benthamiana* leaves were designed to encode proteins with or without carboxy-terminal His-tags; for these alternatives, the same vector was generally used, with or without the natural stop codon of the gene in question. Where the stop codon was removed, an alternative codon (Y) ensured read-through to an additional stretch of 16 amino acids, culminating in six histidine residues (Sainsbury et al. 2009). Leaf samples that had been infiltrated with constructs predicting a His-tagged protein were freeze-dried, ground and extracted in LDS sample buffer (100 μl/mg) (Invitrogen) containing 0.05 M DTT. Samples were analysed on 4–12 % Bis–Tris gels (Novex, Life Technologies), alongside SeeBlue Plus2 markers (Invitrogen). Following electrophoresis, proteins were blotted onto nylon membranes using the Lightning Blot system (Perkin Elmer), membranes were blocked with 3 % BSA in PBST (150 mM NaCl, 10 mM NaPO_4_, pH 7.2 containing 0.5 % Tween 20) for 3 h, and incubated with Anti-6X His tag^®^ antibody [AD1.1.10] (Alkaline Phosphatase) (Abcam, UK) diluted 7:15,000 in 1 % BSA in PBST. Blots were washed three times in PBST, developed using pre-mixed BCIP^®^/NBT solution (SIGMA Aldrich) for up to 15 min, and washed in distilled water to stop development. Duplicate gels were stained for protein using InstantBlue stain (Expedeon) to ensure even loading of samples.

### Evaluation of photosystem complexes following transient expression

Thylakoids were prepared as described by Jarvi et al. (2011), using 5 × 5 mm leaf discs of infiltrated leaf areas per sample. Pelleted thylakoids were suspended in 40 μl native PAGE buffer (Invitrogen) with 1 % *n*-dodecyl-*β*-d-maltoside (Invitrogen) on ice for 15 min, centrifuged for 15 min at 4 °C, and the supernatants removed and stored at −80 °C. Prior to loading gels, 0.75 μl 5 % G-250 sample additive was added to 15 μl aliquots of thylakoid protein extracts. Electrophoresis was carried out using NativePAGE™ 4–16 % Bis–Tris gels (Invitrogen) at 150 V. For two-dimensional analysis, lanes from native PAGE were excised and proteins denatured in LDS sample buffer containing 0.05 M DTT for 30 min. Proteins in gel slices were analysed by electrophoresis using 12 % SDS Bis–Tris two-dimensional well gels (Novex, Life Technologies). Identification of bands was based on comparisons with earlier NativePAGE analytical data (Liu and Last 2015).

### Statistical analysis

Bonferroni tests were used to compare multiple means in datasets, using GenStat 17th Edition, and a significance threshold of *p* < 0.05. Pairwise *t* tests were performed using Excel (*p < 0.05; **p < 0.01; ***p < 0.001).

### Accession of sequence data

Sequence data from this article can be found in the EMBL/GenBank data libraries under accession numbers LN810021 (SGRL genomic, *cv.* Cameor) and LN810020 (SGRL mRNA, *cv.* Cameor).

## Results

### Identification of *SGRL* from pea

As part of the transcriptomic profiling of drought-stressed plants of pea (*Pisum sativum* L.), in which metabolites induced by drought-stress had been identified (Charlton et al. 2008), suppression subtractive hybridisation screening of a cDNA library representing leaf RNA identified 557 genes that were drought-responsive (databases accessions EBI AM161647-AM162203). The 557 sequences represent unigenes in the library, and these have been classified into groups according to likely function (Supplementary Fig. S1a). Representatives of these genes were chosen to examine the ratio of transcript level in drought-stressed compared with well-watered plants across four independent experiments, where a control dehydrin gene (Supplementary Fig. S1b) showed ratios in the range 11–73. Among the cDNAs identified, of those classified as unknown, one partial EST sequence (GenBank accession AM162161) corresponded to a deduced protein sequence that showed low similarity to SGR proteins from a number of species. The entire gene corresponding to the EST sequence was determined for the pea germplasm accession, JI 2822, using gene walking, and the corresponding cDNA isolated from JI 2822 RNA. The limits of the transcribed region were defined, using RNA from the standard cultivar (*cv.*) Cameor, and indicated four transcripts having 5′ untranslated regions of 119, 73, 69 and 53 bases upstream of the start codon. The pea *SGRL* cDNA and genomic sequences are available as GenBank accessions (see “Methods” section).

### Pea *SGRL* represents a distinct class of *SGR* gene

**Fig. 1:**
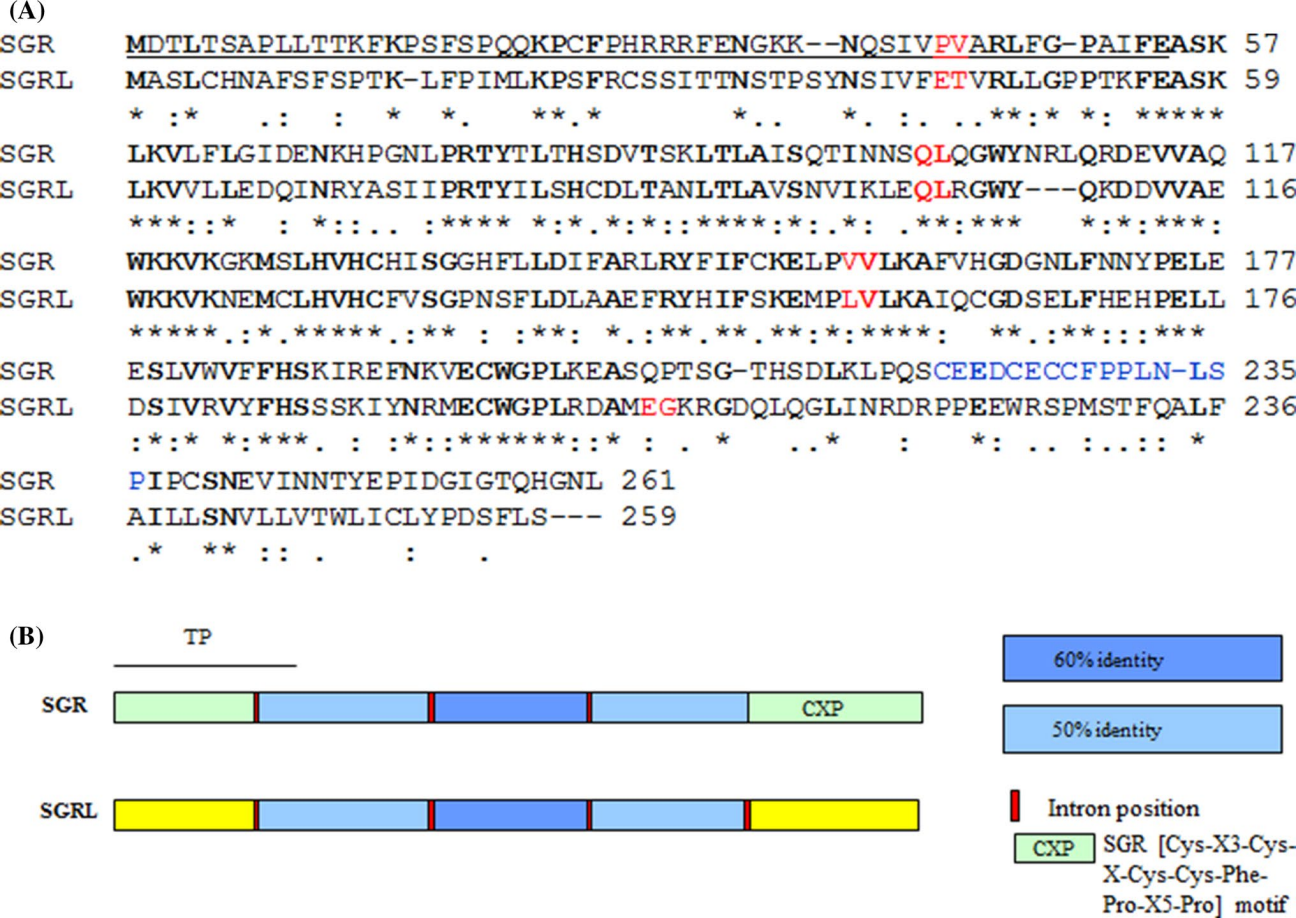
**a** Comparison of the protein sequences deduced for *SGR* and *SGRL* genes from pea. The positions of introns in the corresponding genes are indicated by amino acids in red font, with the predicted transit peptide of SGR *underlined*. The protein sequences show 50 % identity (*bold*, *) and 68 % similarity overall. A fourth intron in *SGRL* and the lack of a [Cys–X3–Cys–X–Cys–Cys–Phe–Pro–X5–Pro] motif (*blue font* in SGR) mark the divergence of SGRL. **b** Schematic of the two proteins, where regions showing more than 50 % identity are *shaded* similarly in blue according to the identities shown. *Yellow* and *pale green* regions indicate regions that are very divergent between the two proteins. The transit peptide region (TP) of SGR is indicated with a *solid line*; CXP indicates the [Cys–X3–Cys–X–Cys–Cys–Phe–Pro–X5–Pro] motif (which is in *blue font* in Fig. 1 **a)**

**Fig. 2:**
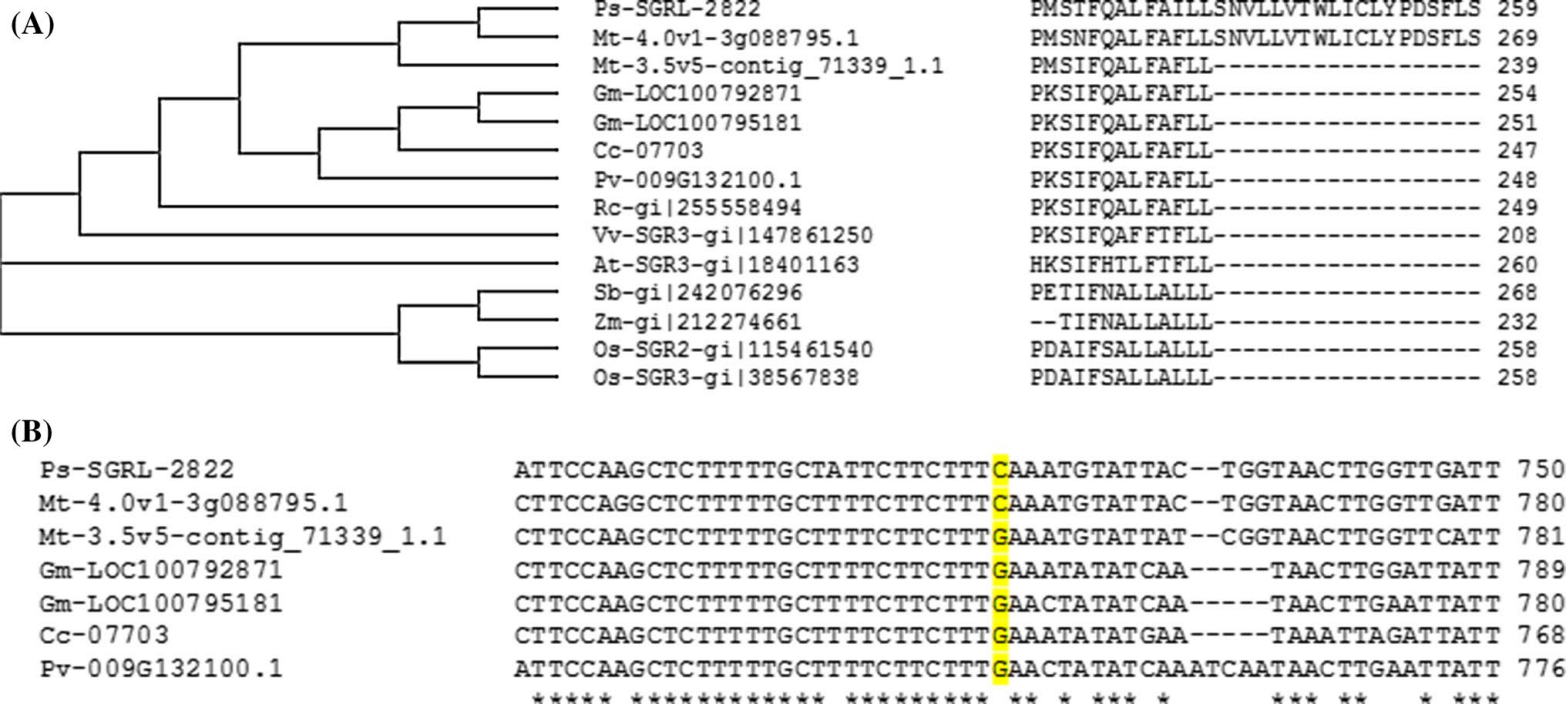
**a** Phylogenetic relationship (*left*), and comparison of carboxy-terminal protein regions (*right*), of the deduced SGRL protein from pea (Ps-SGRL-2822) and related sequences of clade III SGR proteins from other species. Sequences were obtained from Legume Information System (LIS; www.comparative-legumes.org): *Mt* *Medicago truncatula*, *Gm* *Glycine max*, *Cc* *Cajanus cajan*, *Pv* *Phaseolus vulgaris*, *Rc* *Ricinus communis*, *Vv* *Vitis vinifera*, NCBI (www.blast.ncbi.nlm.nih.gov/Blast.cgi): *At* *Arabidopsis thaliana*, *Sb* *Sorghum bicolor*, *Zm* *Zea mays*, *Os* *Oryza sativa,* and aligned using ClustalW2. **b** Comparison of cDNA sequences corresponding to the carboxy-terminal protein regions from the legume sequences used in **a**; *yellow shading* indicates a C>G nucleotide change, compared to Ps-SGRL-2822 and Mt-4.0v1-3g088795.1, that introduces an earlier stop codon in the other genes shown

Sequence data revealed a structure and sequence for *SGRL* which differed markedly from that of *SGR* in pea (Fig. 1a, b). *SGRL* contains four introns, compared with three in *SGR*, and the fourth intron in *SGRL* corresponds to a region beyond which the two sequences diverged significantly. Pea SGRL showed highest similarity to six proteins identified using a BlastP search of the Legume Information System (LIS; www.comparative-legumes.org), where two *Medicago truncatula*, two *Glycine max*, and one each of *Phaseolusvulgaris* and *Cajanuscajan* proteins, were most closely related (Fig. 2). Phylogenetic analysis of SGR proteins across a range of plant genera defined three clades (Aubry et al. 2008). Pea SGRL showed highest similarity to a group of sequences within clade III, which lacks a carboxy-terminal motif (CX_3_CXCCFPX_5_P; Fig. 1) that is highly conserved among clade I and II proteins, representing SGR from dicot and monocot species, respectively (Aubry et al. 2008). Although *SGR* genes in clades I and II are characterised by having three or two introns, respectively, clade III appears to include genes having either three (for example, *Arabidopsis thalianaAtSGR3*) or four (pea *SGRL*, *Vitis viniferaVvSGR3* and two *Glycine max* sequences) introns (data not shown). The pea SGRL protein is distinct from most other clade III members, however, in having an extended carboxy-terminal sequence, compared with related proteins in other species (Fig. 2). One of the two *Medicago* protein sequences identified by LIS also shows this extended carboxy-terminal sequence. Structural predictions (www.cbs.dtu.dk/services/TMHMM/) suggested that the carboxy-terminal region in pea SGRL constitutes a transmembrane domain, a feature also predicted for Mt-4.0v1-3g088795.1 but not for SGR proteins (Supplementary Fig. S2). The remaining clade III members are truncated at a position corresponding to S241 in pea SGRL, which is located centrally within the predicted transmembrane domain. Analysis of cDNA sequences corresponding to this clade of SGR proteins revealed that a C722G nucleotide change relative to pea SGRL resulted in a stop codon in the proteins which are truncated (Fig. 2).

Screening a panel of diverse *Pisum* germplasm accessions and cultivars (19 lines) revealed that the SGRL protein is very highly conserved within *Pisum*. Four exonic SNPs were apparent, all of which are silent with respect to predicted amino acids (T792A in JI 2202; C813T, T840C, A2412G in JI 281, compared with the majority of sequences). In contrast, substantial intronic variation for *SGRL* was observed across the panel; this included a total of 10 insertion/deletions and 38 SNPs (5 and 12, 3 and 9, 2 and 16, and 0 and 1, for insertion/deletions and SNPs in introns 1–4, respectively) (Supplementary Fig. S3a). The variants fell into five categories, for which allele-specific primers facilitated their identification in crosses (Supplementary Fig. S3b). Twelve of the lines formed a group (*cv.* Cameor as type line), with the other seven lines forming a further four categories, where the largest size difference was a 28 bp insertion/deletion in intron 1 of JI 281 (Supplementary Fig. S3a). The ‘Cameor’ and the ‘JI 2822’ groups displayed variation in promoter and 5′ untranslated sequences (three deletions (one bp at −486, eight bp at −322, three bp at 110) and two insertions (one bp at each of −220 and 30, all relative to the start of transcription), plus SNPs were evident in *cv*. Cameor relative to JI 2822 (Supplementary Fig. S3c). These changes were associated with the loss or gain of 14 motifs, according to predictions carried out bioinformatically (http://bioinformatics.psb.ugent.be/webtools/plantcare/html/) on the variant promoter sequences. However, expression of *SGRL* in these two genotypes appeared to be extremely similar (see later).

*SGRL* polymorphisms were exploited to establish that the genomic location of *SGRL* in pea was on linkage group (LG) III, using two mapping populations (JI 281 × JI 399 and Princess × JI 185). In the former, a CAPS marker reflected the smaller intron 1 class in JI 281 (28 bp ‘deletion’ compared with JI 399). This polymorphism mapped very close to *Adh*1 (<1 cM distance, based on *Adh*1 gene scores available for 67 lines) (Supplementary Fig. S4). One nucleotide difference was evident between the *SGRL* promoter region of the parents of a second population, *cv*. Princess and JI 185, which was 790 bp upstream of the start codon. This polymorphism confirmed the linkage group III position obtained for the JI 281 × JI 399 cross. In contrast, *SGR* maps to LG I in pea (data not shown; Armstead et al. 2007).

### *SGRL* and *SGR* show distinct patterns of expression in pea

**Fig. 3:**
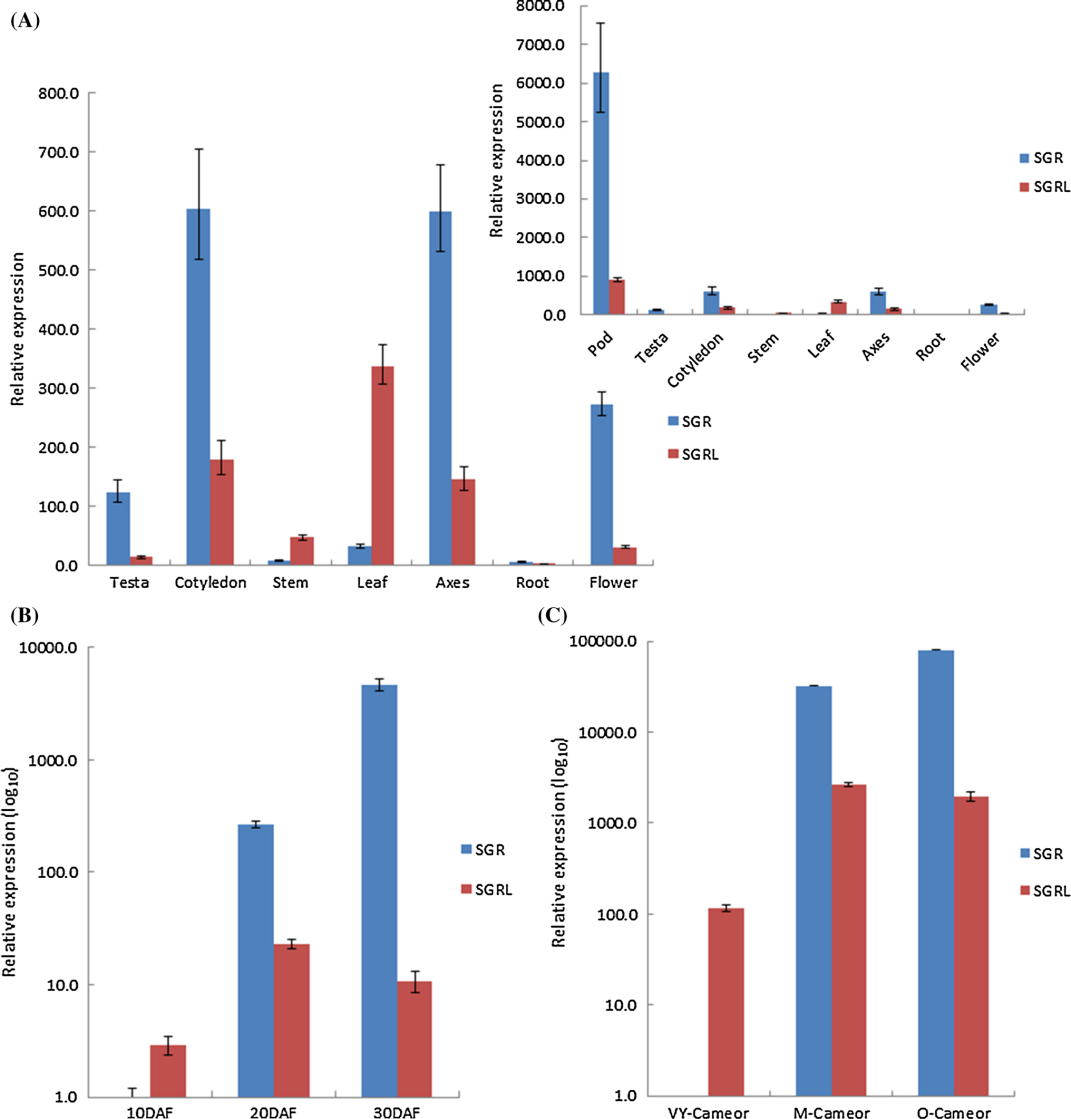

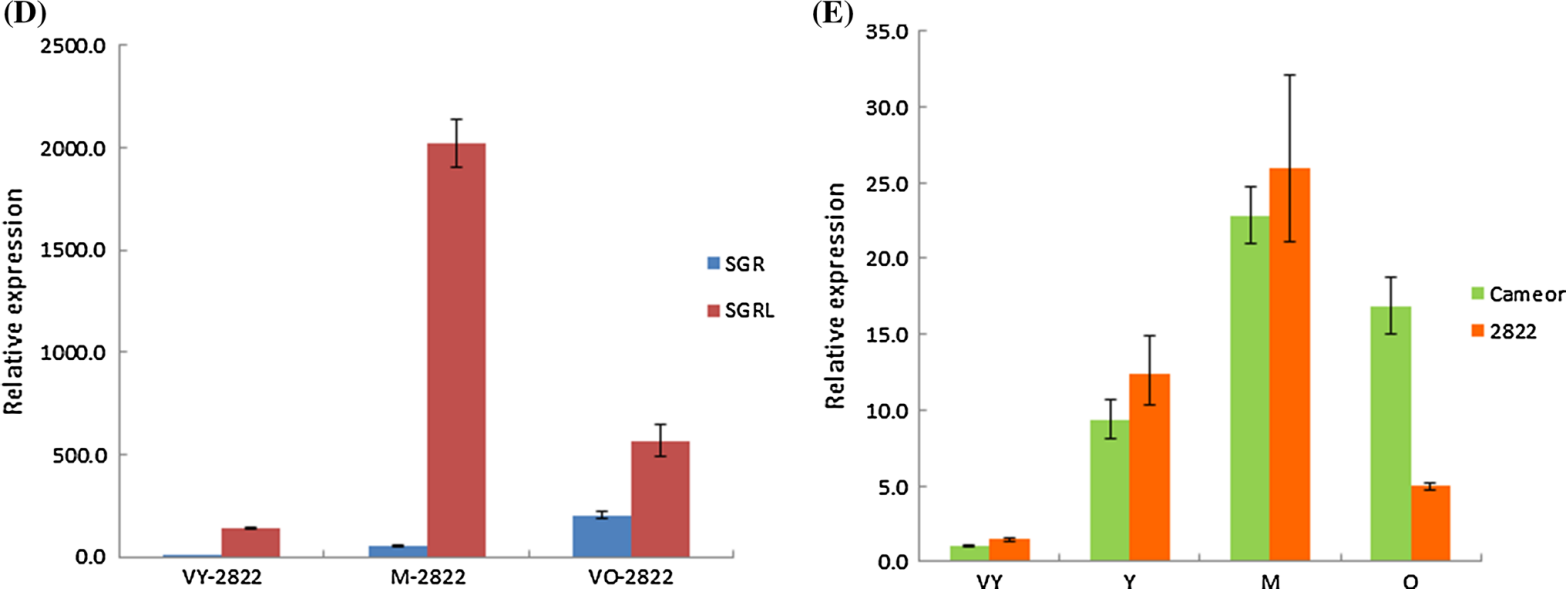
Relative expression of *SGR* and *SGRL* determined by qPCR for **a** Different organs of a wild-type *SGR* genotype (*cv.* Birte). Comparisons of expression are shown for testa, cotyledon, stem, leaf, axes, root and flower (main graph), with the much higher relative expression of *SGR* in pods shown in inset graph, using *SGRL* root expression as the calibrator. **b** Embryos (cotyledons with axes) at 10, 20 and 30 days after flowering (DAF) from a wild-type *SGR* genotype (*cv.* Birte), displayed on a log_10_ scale using *SGR* 10 DAF expression as the calibrator. **c** Leaves from wild-type *SGR* plants at three stages of development (*VY* very young, *M* mature, *O* senescing, *cv.* Cameor), displayed on a log_10_ scale using *SGR* VY expression as the calibrator. **d** Leaves from a mutant *sgr* genotype (*VY* very young, *M* mature, *VO* senescing, JI 2822), using *SGR* VY expression as the calibrator. **e** Relative expression of *SGRL* at four stages of leaf development in a wild-type *SGR* (*cv.* Cameor) or mutant *sgr* (JI 2822) genotype (*VY* very young, *Y* young, *M* mature, *O* senescing), using *cv.* Cameor VY expression as the calibrator

RT-PCR analysis showed that both *SGRL* and *SGR* were expressed in all major plant organs of pea (not shown). Analysis of expression by qPCR revealed that *SGRL* expression was lower than that of *SGR* in all organs, apart from stem and young leaf (Fig. 3a). The expression of *SGR* was highest in reproductive organs (pods, seeds and flowers) compared with other plant parts, whereas expression of *SGRL* was highest in pods and leaves. For both genes, roots showed the lowest expression level, with relative expression ranked as: pod > leaf > cotyledon = axes > stem > flower > testa > root for *SGRL*, compared with pod > cotyledon = axes > flower > testa > leaf > stem > root for *SGR* (Fig. 3a).

Further quantitative analysis of *SGR* and *SGRL* gene expression during development was carried out using genotypes that were wild-type (*cv.* Birte or *cv.* Cameor, yellow cotyledon phenotype) or mutant (JI 2822, green cotyledon phenotype) for *SGR*. The *sgr* allele in JI 2822 (data not shown) belongs to a class of non-coding mutations described by Sato et al. (2007), where *SGR* transcripts are detected. Analyses throughout seed development in the wild-type *SGR* lines showed that, whereas *SGR* expression increased dramatically between 20 and 30 days after flowering (DAF), the expression of *SGRL* was maximal at 20 DAF (Fig. 3b). Expression of *SGRL* was higher than that of *SGR* at 10 DAF, it was lower at 20 DAF, while at 30 DAF it was very low in comparison with the very high expression of *SGR* (Fig. 3b).

Quantitative analysis of *SGR* and *SGRL* expression throughout leaf development in the wild-type *SGR* background showed that *SGR* expression increased throughout leaf development with much higher expression in mature and senescing leaves (Fig. 3c). In contrast, expression of *SGRL* increased at first but not at senescence. Overall, *SGRL* showed much lower expression levels than *SGR*, except in the youngest leaves. The quantitative expression of *SGRL* during leaf development was very similar in the *sgr* mutant genotype, JI 2822 (Fig. 3d, e). Although a lower expression was determined for the most advanced leaves of JI 2822 compared with those of *cv.* Cameor, this is likely to reflect apparent differences in the stages of senescence, due to JI 2822 (*sgr*) having a stay-green phenotype. Despite the considerable variation in the promoter regions and promoter motif predictions that was noted for *SGRL* in *cv.* Cameor and JI 2822 (Supplementary Fig. S3c), the expression data suggested very similar expression profiles for the variant genes. The data further indicated that *SGRL* expression was independent of that of *SGR*.

### Transient expression of pea *SGRL* and *SGR* genes in *Nicotiana benthamiana* leaves triggers chlorophyll metabolism

**Fig. 4:**
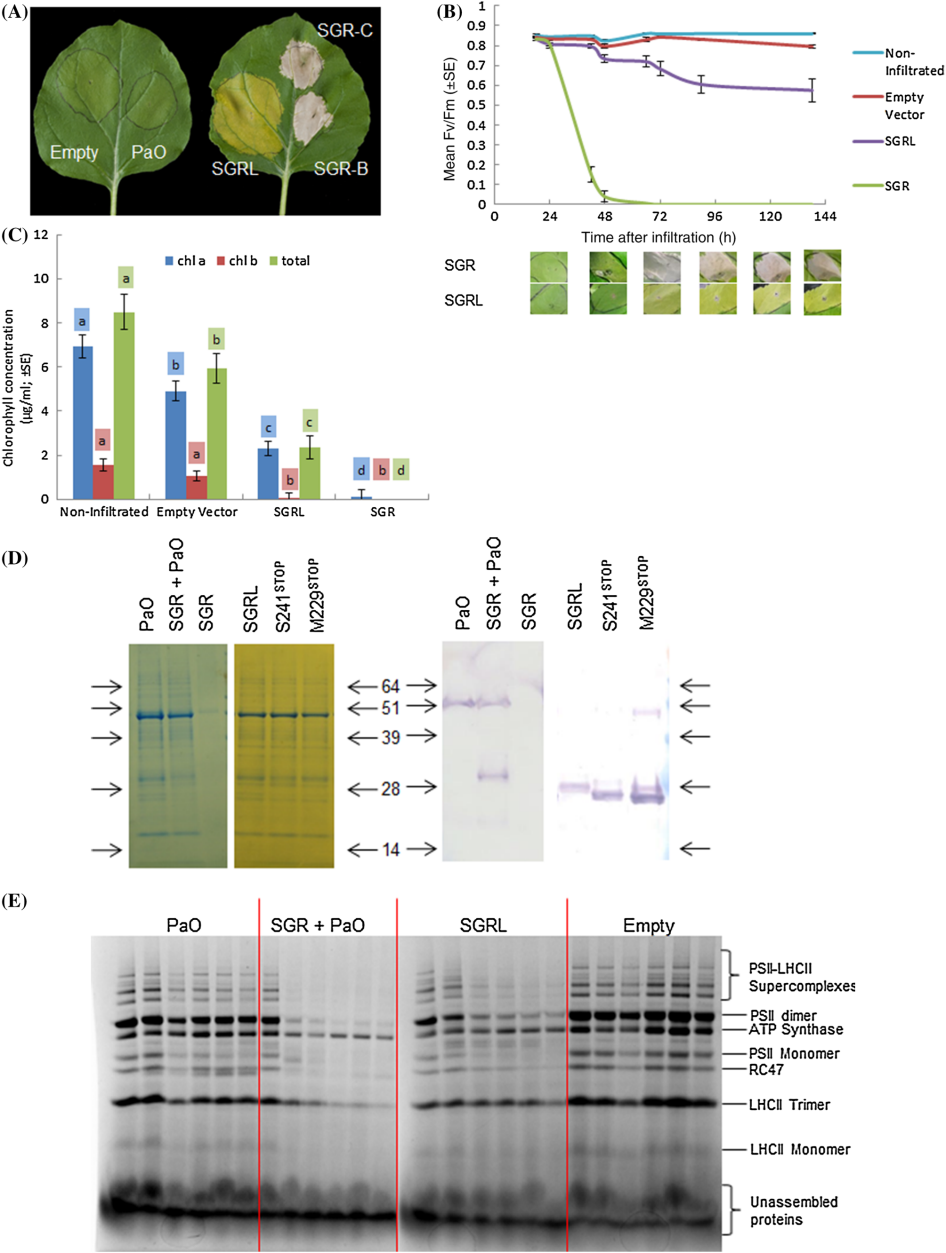
**a** Phenotypes of leaves of *Nicotiana benthamiana* showing effects of transiently expressed gene constructs: Empty (Empty Vector) and *PaO* (*left*) and *SGRL* and two wild-type alleles of *SGR* (*right*) after 144 h; *SGR*-*C*, *SGR* from *cv*. Cameor; *SGR*-*B*, *SGR* from *cv*. Birte. **b** Changes in Fv/Fm over time following infiltration of *SGRL*, *SGR* or Empty Vector constructs in comparison to non-infiltrated areas, with images of leaves at each time point shown below. **c** Chlorophyll (*a*, *b* and total) concentration of leaf areas following transient expression for 140 h (see **b**), with individual Bonferroni statistical analysis for chlorophyll *a*, *b* and total as indicated by *highlighted letters*; within each analysis, *different letters* (**a**–**d**) indicate significant differences; *p* < 0.05. **d** Analysis of transiently expressed proteins 144 h after infiltration of *PaO, SGR, SGR* plus *PaO, SGRL* or its mutant derivatives, S241^stop^, M229^stop^; gels showing total proteins stained (*left two panels*), blots showing His-tagged proteins (*right two panels*). *Arrows* indicate the positions of protein size markers, with their molecular weights indicated in the centre (kDa × 10^−3^). **e** Non-denaturing PAGE analysis of proteins from leaf areas infiltrated with *PaO, SGR* plus *PaO, SGRL* and Empty (Empty Vector) at 24, 48, 72, 96, 120 and 144 h after infiltration (*left* to *right* in every panel); the positions of the major photosystem proteins according to Liu and Last (2015) are indicated on the right hand side

In order to investigate the function of SGRL, transient expression of SGRL and SGR from pea was carried out, using agro-infiltration of *Nicotiana benthamiana* leaf tissue (Sainsbury et al. 2009). Full-length cDNA sequences were cloned into pEAQ-*HT* DEST1 and DEST3 vectors to enable high-level expression of native and His-tagged proteins, respectively. Expression of SGRL showed a clear loss of green colour phenotype in leaf tissue within days of infiltration, whereas control constructs (empty vector, *GFP* or other genes that participate in the chlorophyll degradation pathway, for example, *PaO*) did not induce such a phenotype (Fig. 4a). Transient expression of SGRL produced a phenotype which differed markedly from that of the related SGR, for which an early yellowing phenotype was observed followed by whitening of the tissue (Fig. 4b). The phenotype induced by SGR was consistent with that reported for *pao*-deficient mutants of *Arabidopsisthaliana* and *Zea mays* (Yang et al. 2004) and in SGR-overexpressing lines of *Arabidopsis thaliana*, where it was concluded that SGR effects and absence of PaO were linked to stresses leading to a hypersensitive cell-death response (Mur et al. 2010). The SGR- and SGRL-induced phenotypes (Fig. 4a) were consistent over replicated transient expression experiments, and their contrasting visual appearance was apparent within 24 and 36 h following infiltration. The relative losses of chlorophyll were monitored, using measurements of photosynthetic efficiency and chlorophyll concentration in leaf discs. The photosynthetic ability of infiltrated leaf areas, based on Fv/Fm determinations, provided a measure of photosystem II efficiency. These data (Fig. 4b) showed that, while SGR promoted a rapid loss of photosynthetic efficiency within 48 h, the effect of SGRL expression was less severe, with Fv/Fm declining more slowly and plateauing at around 90 h post-infiltration. Both SGR and SGRL led to a reduction in total chlorophyll compared with non-infiltrated and empty vector-infiltrated leaf areas (Fig. 4c). SGRL appeared to cause a preferential decrease in chlorophyll *b*, while chlorophyll *a* also decreased in concentration. In contrast, SGR showed no preference for either chlorophyll, leading to its complete loss.

The phenotypes observed for individual constructs were consistent across many experiments and were reproducible, whether specific gene constructs encoded His-tagged proteins or not. Western blots of infiltrated leaf areas expressing His-tagged SGRL and other His-tagged proteins implicated in the chlorophyll degradation pathway showed that all could be detected up to 168 h after infiltration, with the exception of SGR (see Fig. 4d). The rapid ‘cell-death’ phenotype of leaves expressing SGR was associated with a complete loss of leaf protein, as shown by SDS-PAGE (Fig. 4d). In an attempt to alleviate the SGR-induced phenotype, a likely consequence of high-level accumulation of phototoxic chlorophyll intermediates (Yang et al. 2004; Mur et al. 2010), and thereby facilitate the detection of expressed proteins, co-expression with PaO was carried out. In these co-expression assays, whitening of the leaf tissue was not observed throughout 144 h post-infiltration (Supplementary Fig. S5a), while the loss of chlorophyll at 144 h post-infiltration was similar to that for SGR alone (Supplementary Fig. S5b). Interestingly, at an earlier time point (72 h post-infiltration), Fv/Fm values were higher for the double-infiltrated leaf areas than for SGR alone, likely due to the absence of the apparent cell-death response (Supplementary Fig. S5c). Figure 4d shows the detection with an anti-His antibody of a range of transiently expressed proteins, including SGR, PaO, SGRL and its derivatives, S241^STOP^ and M229^STOP^, discussed below. The expressed proteins showed mobilities that were in agreement with their expected masses, including the carboxy-terminal extension (see “Methods” section).

Non-denaturing analysis of leaf protein complexes was performed to analyse the effects of SGR and SGRL transient expression in more detail (Fig. 4e). As shown above, co-infiltration of *PaO* with *SGR* was necessary for protein analysis in *SGR*-infiltrated leaves (see Fig. 4d). After 48 h, while *PaO* alone did not induce any substantive change in the protein profile overall, leaf samples infiltrated with *SGR* and *PaO* showed a loss of all PSII-LHCII supercomplexes (Fig. 4e). After 72 h, PSII dimers were barely detected in *SGR* plus *PaO*-infiltrated leaves, while ATP synthase and LHCII trimers could be detected through to 144 h, with ATP synthase showing highest stability. SGRL induced a similar but more gradual loss of PSII-LHCII supercomplexes. Here, however, PSII dimers and LHCII trimers were apparent through to 144 h post-infiltration. *PaO*- and empty vector-infiltrated leaf areas showed no differences in protein complexes, except for an additional protein evident in the former, which migrated close to the RC47 band. Western blot analysis following non-denaturing gel separation revealed that the additional protein corresponded to His-tagged PaO (Supplementary Fig. S5d), with a molecular weight on denaturing second-dimensional gels that was consistent with its predicted size (57.1 × 10^−3^ kDa, Supplementary Fig. S5e).

### Pea *SGRL* and *SGR* genes show distinct modes of action under light and dark conditions and do not function cooperatively

**Fig. 5:**
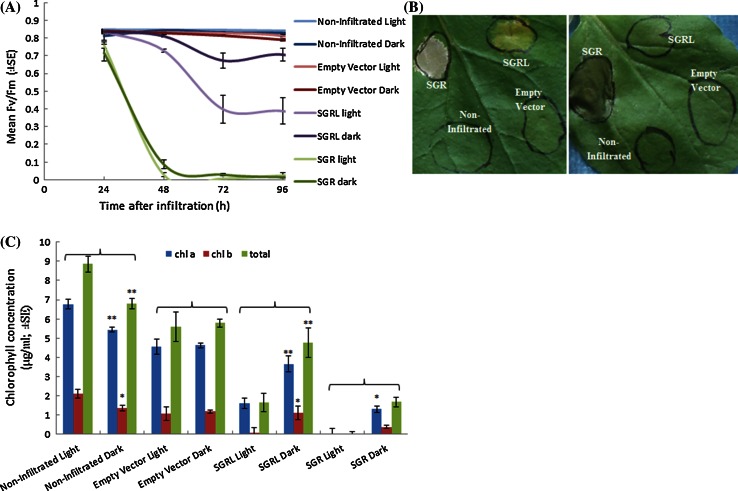
**a** Changes in Fv/Fm over time in leaves following infiltration of *SGRL*, *SGR* or Empty Vector constructs in comparison to non-infiltrated areas, maintained in ‘light’ or ‘dark’ conditions. **b** Phenotype of leaves showing effects of transiently expressed constructs (as in **a**), under ‘light’ (*left*) or ‘dark’ (*right*) conditions 144 h after infiltration **c** Chlorophyll concentration (*a*, *b* and total) of leaf areas following transient expression (see **a**) for 140 h. Pairwise *t* tests indicated significant differences in chlorophyll concentration between light**-** and dark**-**incubated samples (*p < 0.05, **p < 0.01) within the constructs and controls tested, as indicated by *brackets*

The action of SGRL was explored further in experiments carried out in either ‘light’ (regular day/night cycling) or ‘dark’ conditions (plants deprived of any light from the point of infiltration until the end of the experiment). Under the ‘dark’ conditions, SGR expression led to a rapid loss of photosynthetic ability, but without displaying the leaf whitening or ‘cell-death’ phenotype, in agreement with the light-induced phototoxicity of some chlorophyll pathway intermediates (Yang et al. 2004; Mur et al. 2010) (Fig. 5a, b). Under the ‘dark’ conditions, SGRL expression showed a much lower ability to reduce Fv/Fm compared with areas exposed to the ‘light’ regime (Fig. 5a); furthermore, under the ‘dark’ conditions, SGRL expression did not lead to the leaf yellowing phenotype seen under ‘light’ conditions (Fig. 5b). The characteristic total loss of chlorophyll for SGR was observed under ‘light’ conditions, and a severe reduction in total chlorophyll under ‘dark’ conditions. In agreement with Fv/Fm measurements, leaf areas expressing SGRL differed greatly in ‘light’ and ‘dark’ comparisons. Under ‘light’ conditions, SGRL expression led to the expected loss in chlorophyll *b* (see Fig. 4c) but, under ‘dark’ conditions, a significantly lower loss of chlorophyll (*a*, *b* and total), compared with ‘light’ conditions, was observed (Fig. 5c).

**Fig. 6:**
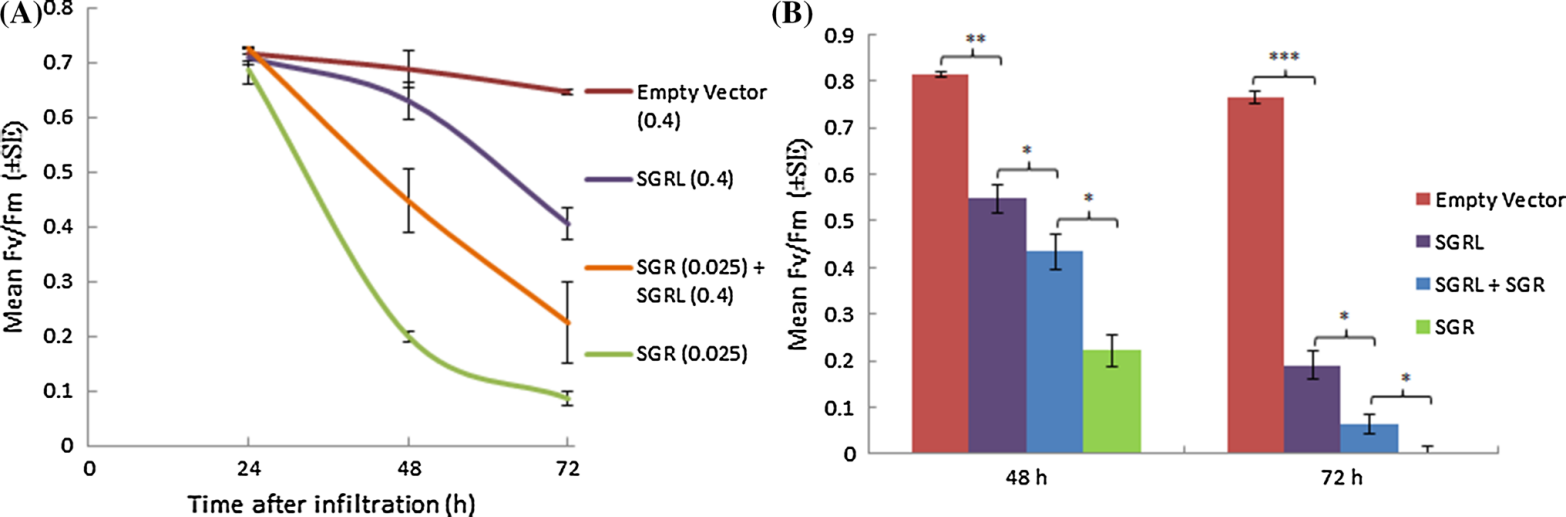
**a** Changes in Fv/Fm over time in leaves following infiltration of *SGRL*, *SGR*, Empty Vector or *SGR* plus *SGRL*, using constructs at OD values shown. **b** Fv/Fm values following infiltration of *SGRL*, *SGR*, Empty Vector or *SGR* plus *SGRL* at 48 and 72 h post-infiltration, where *SGR* constructs were at 0.03 OD. Significance values determined by *t* tests are shown for pair-wise comparisons, as indicated by *brackets* (*p < 0.05; **p < 0.01; ***p < 0.001)

The extent to which SGR and SGRL might function cooperatively or otherwise to coordinate chlorophyll degradation was investigated by performing co-infiltrations of the two constructs into leaves of *Nicotiana benthamiana*. These experiments necessitated the infiltration of *Agrobacterium* harbouring SGR constructs at much lower cell densities, in order to reduce the ‘cell-death’ phenotype typically associated with SGR (see Fig. 4a, d), and to reduce the rate of corresponding loss of Fv/Fm (Fig. 4b). Co-infiltration experiments, involving adjustment of one or more Agrobacterium cultures to a standard OD value, were validated in comparisons involving a co-infiltrated unrelated gene. The results showed that a co-infiltrated unrelated gene (*GFP*) had no significant effect on the phenotypes observed for either *SGR* or *SGRL*, in contrast to the data obtained for the combination of *SGR* and *SGRL*; these data and their statistical analyses are presented in Supplementary Fig. S6. Co-infiltration of *SGRL* with *SGR* (the latter at low OD_600_) showed that SGRL expression did not enhance the ability of SGR to reduce photosynthetic efficiency, and intermediate Fv/Fm values were measured up to 72 h post-infiltration; while the values were lower than those obtained for SGRL, they were higher than those for SGR controls (Fig. 6a). Infiltration of *SGR* with and without *GFP* led to significantly lower Fv/Fm values at both 48 and 72 h after infiltration, compared to the combination of *SGR* with *SGRL*, while *SGRL* with or without *GFP* led to higher levels of Fv/Fm compared to co-infiltrated *SGRL* and *SGR* (Fig. 6b, Supplementary Fig. S6). At the lower concentrations used here, SGR expression led to the characteristic ‘cell-death’ phenotype but at a much slower rate (72 h post-infiltration (Supplementary Fig. S7). Co-infiltration of *SGRL* and *SGR* not only led to higher values for Fv/Fm being maintained (Fig. 6) but also delayed the onset of cell death to 120–144 h post-infiltration (Supplementary Fig. S7).

### Transient expression of mutant *SGRL* sequences reveals functional domains

**Fig. 7:**
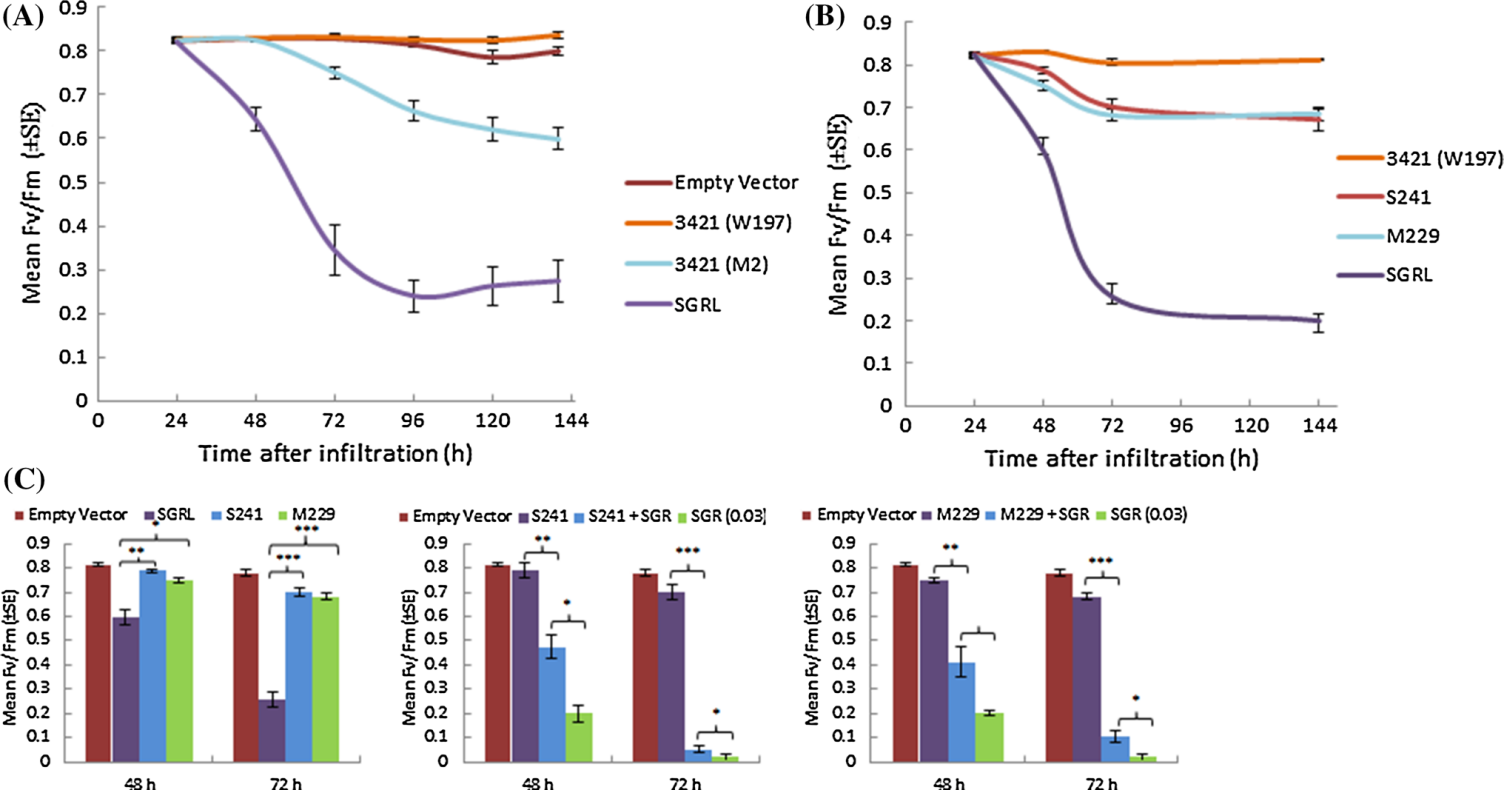
**a** Changes in Fv/Fm over time in leaves following infiltration of *SGRL* or mutant *sgrL* constructs; 3421 (W197), a TILLING mutant *sgrL* encoding a truncated protein (W197^STOP^) and 3421(M2) derived from 3421. **b** Changes in Fv/Fm over time following infiltration of *SGRL*, its derivative constructs: S241 (S241^STOP^) and M229 (M229^STOP^), or the *sgrL* mutant 3421 (W197^STOP^). **c** Fv/Fm values determined, following infiltration of *SGR* (OD_600_ 0.03), *SGRL* or its derivatives, S241 (S241^STOP^) and M229 (M229^STOP^), in combinations with *SGR* (OD_600_ 0.03), at 48 and 72 h post-infiltration. Significance values determined by *t* tests are shown for pair-wise comparisons, as indicated by brackets (*p < 0.05, **p < 0.01, ***p < 0.001)

The function of SGRL in vivo was investigated in induced mutants of pea (*cv*. Cameor) identified by TILLING (http://www-urgv.versailles.inra.fr/tilling/index.htm; Dalmais et al. 2008). A mutation predicted to have major consequences for protein function was used in the subsequent studies: *sgrL*^*3421*^ (W197^STOP^), where an early stop mutation is encoded at the W197 position. Homozygous mutant seeds were obtained for this mutation, which did not give rise to cotyledons having a stay-green phenotype (not shown), unlike *sgr* mutants. Transient expression assays using the mutant *SGRL* gene suggested that the *sgrL*^*3421*^ (W197^STOP^) variant was a loss-of-function mutant (Fig. 7a). During cloning, a variant of this mutant construct (*sgrL*^*3421 (M2)*^) was generated, in which a section of 120 bp and amino acids D165–M204 (inclusive of W197^STOP^) were lost, but the reading frame was restored beyond the deleted section (Fig. 1a; see Supplementary Fig. S8). This mutant construct maintained some function in transient expression assays, albeit at a reduced level compared with that of the wild-type *SGRL* (Fig. 7a), but it did not lead to loss of green colour in leaves (Supplementary Fig. S9). In order to establish functional domains for SGRL, further mutant sequences were engineered to have induced premature stop codons in two carboxy-terminal regions; the first of these was positioned just before the predicted transmembrane domain (M229^STOP^) and the second (S241^STOP^) to mimic the truncated SGRL proteins in other species (Fig. 2; see Supplementary Fig. S8). These last two mutant constructs showed reduced functionality compared to pea *SGRL*, but retained some activity in contrast to that of the loss-of-function TILLING mutant allele, W197^STOP^ (Fig. 7b, Supplementary Fig. S9). Neither of the two engineered SGRL truncated proteins was capable of enhancing the action of SGR, with effects that were similar to those determined for SGRL on SGR (Fig. 6a, b), as shown at 48 and 72 h following infiltration (Fig. 7b, c). Here, co-infiltration of truncated *SGRL* constructs with *SGR* reduced Fv/Fm considerably compared to truncated *SGRL* constructs, but the values determined for the combinations were significantly higher than those for SGR alone (Fig. 7c). Furthermore the onset of the ‘cell-death’ phenotype in *SGR* and co-infiltrated leaf areas followed a similar pattern to that of *SGRL* co-infiltrations, being considerably delayed (Supplementary Fig. S7).

### Mutations that impair the function of SGRL impact negatively on photosynthetic capacity and growth

**Fig. 8:**
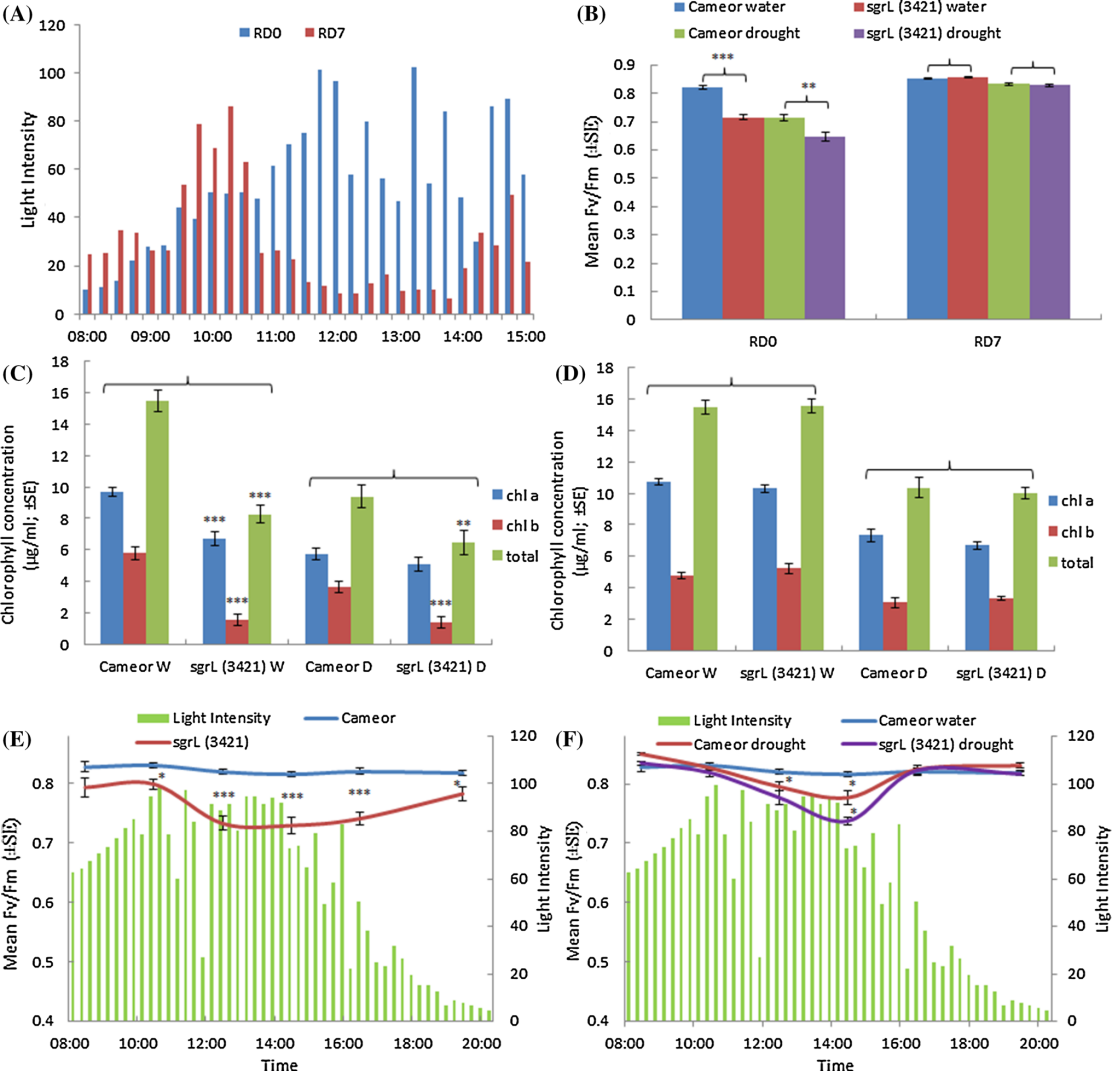
Comparisons of photosynthetic efficiency (mean Fv/Fm) and chlorophyll concentration in *sgrL* ^*3421*^ mutant and *cv.* Cameor (control) plants under well-watered or drought-stressed conditions with significance values determined by *t* tests, as indicated by *brackets* (**p < 0.01, ***p < 0.001) **a** Light intensities (kilolux) during the day at the times shown for two contrasting days, RD0 (*blue bars*) and RD7 (*red bars*). **b** Mean Fv/Fm of *cv.* Cameor and *sgrL* ^*3421*^ mutant plants under well-watered or drought-stressed conditions for RD0 and RD7 taken at 13:30 each day. **c**, **d** Chlorophyll (*a*, *b* and total) concentration of leaf areas of *cv.* Cameor and *sgrL* ^*3421*^ mutant plants under well-watered (W) or drought-stressed (D) conditions for RD0 (**c**) and for RD7 (**d**). **e**, **f** Change in Fv/Fm over 12 h of one day (RD13) for well-watered *sgrL* ^*3421*^ (*red*) and well-watered *cv.* Cameor (*blue*) (**e**) and well-watered *cv.* Cameor (*blue*), drought-stressed *cv.* Cameor (*red*) and drought-stressed *sgrL* ^*3421*^ (*purple*) (**f)** plants, where relative light intensity (kilolux, right-hand scale) is shown as green bars; both sets of drought-stressed plants received 20 ml water per plant at 14:30. Significance values were determined by *t* tests (*p < 0.05, ***p < 0.001) and indicate differences between the data points indicated

The impact of the *SGRL* mutation in *sgrL*^*3421*^ (W197^STOP^) plants was investigated in both drought-stressed and well-watered conditions, by monitoring changes in photosynthetic ability (Fv/Fm) and chlorophyll concentration. Light intensity data were recorded for the days when Fv/Fm measurements were recorded. These data revealed much higher light intensities on RD0 compared with RD7, especially in the time period leading up to measurement and sampling on those days (13:30; Fig. 8a). Photosynthetic efficiency of the mutant plants was significantly reduced compared to the wild type (*cv.* Cameor) on RD0 (Fig. 8b), which showed high light intensity (Fig. 8a), while in contrast no difference in photosynthetic efficiency was observed under lower light intensity (RD7) (Fig. 8a, b). Differences in chlorophyll concentration supported the Fv/Fm data, with *sgrL*^*3421*^ mutants having lower chlorophyll compared to the wild type on RD0 (Fig. 8c), while on RD7 there were no significant differences between the groups (Fig. 8d).

Changes in photosynthetic efficiency (Fv/Fm) of *sgrL*^*3421*^ mutant and *cv.* Cameor plants were monitored across RD13 when light intensity was high throughout the bulk of the day (Fig. 8e, f). Photosynthetic efficiency dropped dramatically in well-watered *sgrL*^*3421*^ plants as light intensity peaked and remained significantly lower than *cv.* Cameor, while showing a recovery when light intensity reduced after 16:00 (Fig. 8e). Drought-stressed plants showed reduced Fv/Fm as light intensity increased, with the *sgrL*^*3421*^ mutant plants showing a more significant drop. After the drought-stressed plants were watered and as light intensity reduced, their photosynthetic efficiencies recovered to values that were similar in the well-watered control wild-type plants (Fig. 8f).

**Fig. 9:**
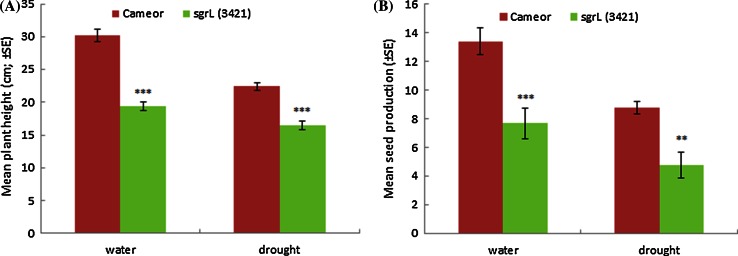
Comparisons of *sgrL* ^*3421*^ mutant plant phenotypes with those of *cv.* Cameor (control); significance values determined by *t* tests of data for *cv.* Cameor and mutant plants (**p < 0.01, ***p < 0.001) **a** Mean plant height (cm) of *cv.* Cameor and *sgrL* ^*3421*^ mutant under well-watered or drought-stressed conditions. **b** Mean seed production per plant of *cv.* Cameor and *sgrL* ^*3421*^ under well-watered or drought-stressed conditions

Phenotypic trait measurements of *sgrL*^*3421*^ (W197^STOP^) mutant and control plants revealed highly significant differences between the responses of mutant and wild-type lines (Fig. 9). In the mutants, the overall height of mature plants was reduced under both well-watered and drought-stressed conditions (by an average of 10.87 ± 1.16 and 5.93 ± 0.86 cm, respectively); mutant plants also yielded fewer seeds under the two conditions (an average of 5.7 ± 1.40 and 4 ± 1.01 fewer seeds, respectively) (Fig. 9a, b) and had a much weedier habit overall than the control plants. The difference in height and overall vigour of well-watered and drought-stressed plants is shown in Supplementary Fig. S10, along with plant height data, determined from a repeat experiment. Here a significant difference (*p* < 0.001) was also observed for plant height in control compared to mutant plants under these different growth conditions when absolute values differed overall. Mutant plants were shorter by an average of 18.67 ± 4.04 cm in well-watered conditions and by an average of 6.03 ± 1.18 cm under drought-stressed conditions. In this second growth experiment, plants were handled extensively for measurements of photosynthetic efficiency and sampled for chlorophyll measurements, and therefore seed production data for these plants are not presented here.

## Discussion

This work describes a novel and distinct SGR protein (SGRL) in pea and its functional properties, determined from analysis of wild-type and mutant proteins, which indicate a role that differs from that of SGR. Pea SGRL has several features that distinguish it from the senescence-induced regulator of chlorophyll degradation, SGR (Park et al. 2007; Hӧrtensteiner et al. 2009). The structure of pea SGRL is distinguished primarily by its divergent carboxy-terminal region, and lack of a domain that is highly conserved among SGR proteins (Fig. 1a). The distinct expression pattern of pea *SGRL* compared to *SGR* (Fig. 3b, c) suggests that SGRL is more likely to play a role in earlier plant development, in contrast to SGR. Most SGR proteins have been reported to be induced during senescence; exceptions are SGRL in rice and *Arabidopsis thaliana*. In rice, a higher expression of *SGRL* in green tissues and a reduction of expression during senescence have been reported (Rong et al. 2013), in agreement with the expression data for pea *SGRL* reported here. Expression patterns of *Arabidopsis thaliana SGRL* have been reported to be higher in developing and pre-senescent tissue, before declining during senescence, and furthermore to be down-regulated during dark incubation (Sakuraba et al. 2014b).

It has been suggested that SGR controls chlorophyll degradation by interacting directly with LHCII, by recruiting the major chlorophyll degradation enzymes, or a combination of the two processes (Sakuraba et al. 2012). The clear but limited similarity between SGRL and SGR suggested that the former may contribute to chlorophyll catabolism. The possible role for SGRL in the processes associated with chlorophyll degradation was investigated, using a set of induced and artificially engineered mutants in in vivo and in vitro (transient expression) assays, respectively. The assays exploited vectors that were optimised to promote high-level gene expression in leaves of *Nicotiana benthamiana* (Sainsbury et al. 2009), and were demonstrated in this work to be robust and reproducible; control genes and vectors alone elicited no response in terms of the phenotypic or biochemical traits being measured. It was also clear from assays of pea SGR alone that further enzymatic steps of the pathway were initiated in *Nicotiana* and that the ‘cell death’ phenotype that was evident under light conditions could be alleviated by co-expression of *PaO* with *SGR*, in agreement with the role of PaO in the removal of phototoxic intermediates of the pathway (Yang et al. 2004). In the assays described in this work, the phenotype obtained with *SGRL* alone was distinctly different from that of *SGR* and the ‘cell death’ phenotype was never observed in assays of the former.

In the investigations reported here, while SGR was capable of promoting the breakdown of chlorophyll in both ‘light’ and ‘dark’ conditions, SGRL was associated with the preferential loss of chlorophyll *b* in a light-dependent manner (Fig. 5). It has been suggested that chlorophyll *b* breakdown is catalysed by a complex involving NOL, which is anchored to the thylakoid membrane by NYC1 and an unknown ‘factor x’ (Sato et al. 2009). Due to the ability of SGRL to preferentially promote the loss of chlorophyll *b* in this study, SGRL with its predicted transmembrane domain may be a candidate for the proposed membrane-anchored ‘factor x’. The complex involving NYC1 has also been suggested to play a role in the breakdown of LHCII which contains chlorophyll *a* and *b* (Kasuba et al. 2007; Horie et al. 2009); however, no explanation has been provided hitherto as to how the whole photosystem is degraded. In rice, it has been shown that SGRL, distinct from SGR, can bind to LHCII, and that SGRL overexpression lines also show lower levels of LHCII (Rong et al. 2013). In *Arabidopsis thaliana*, SGRL has been shown to co-immunoprecipitate with LHCII (Sakuraba et al. 2014a). Evaluation of protein complexes following transient expression showed that both pea SGR and SGRL led to a reduction of PSII-LHCII supercomplexes; however, SGR also led to greater loss of PSII dimers and monomers (Fig. 4e). This suggests that SGRL is capable of interacting with and degrading supercomplexes by removing outer subunits, while leaving core subunits intact. This hypothesis is supported by chlorophyll measurements; outer subunits of the supercomplexes contain proteins capable of binding both chlorophyll *a* and *b*, whereas central PSII core subunits contain only chlorophyll *a*-binding proteins (Hankamer and Barber 1997). The data presented here (Fig. 5c) show that, in leaves where SGRL is transiently expressed, some chlorophyll *a* is retained whereas chlorophyll *b* is completely lost.

Both pea SGR and SGRL led to a reduction in Fv/Fm and a loss of green colouration and chlorophyll (Fig. 4b, c), presumably by initiating LHCII dismantling (Rong et al. 2013; Sakuraba et al. 2014a; Horie et al. 2009; Kasuba et al. 2007), and both showed the capacity to degrade PSII-LHCII supercomplexes (Fig. 4e). Therefore the possibility that the two proteins might act cooperatively in these processes was investigated. The data strongly suggested, however, that SGRL reduced the SGR-induced loss of chlorophyll and Fv/Fm, as a measure of photosynthetic ability, compared with SGR alone, indicating a non-cooperative action (Fig. 6a, b). In combination with expression patterns during development, the data suggested that SGRL may play a role in chlorophyll turnover or re-cycling during plant growth and development, supported by the light-dependent nature of the transient expression assay results (Fig. 5). Turnover of chlorophyll from potentially damaged photosystems would be required mostly in daylight when photosystems are being utilised and damaged. Expression patterns for rice *SGRL* were noted to change over the course of the day, with maximal expression in the morning declining to minimal in the evening (Rong et al. 2013). A study in *Arabidopsis thaliana* has indicated that, under stress conditions, plants overexpressing SGRL displayed a yellowing phenotype whereas, under dark incubation, this phenotype was much reduced (Sakuraba et al. 2014a). Furthermore, it was shown in *Arabidopsis thaliana* that *SGRL* expression is greatly diminished during dark incubation (Sakuraba et al. 2014b).

The idea of a chlorophyll recovery pathway arose from radioactive labelling studies, which showed that the majority of chlorophylls found in LHCII of a cyanobacterium (*Synechocystis* species) had originated from re-cycled chlorophyll molecules (Kopecná et al. 2012). A chlorophyll salvage pathway has also been suggested for *Arabidopsis thaliana* and, although a mechanism for this has yet to be elucidated, chlorophyllide *a* appears to be the point at which chlorophyll turnover products re-enter the synthesis pathway (Lin et al. 2014; Balazadeh 2014). Due to similarities in the absorbance spectra of chlorophyllide and chlorophyll, both are detected in the chlorophyll measurements presented here (Hu et al. 2013; Milenković et al. 2012), potentially contributing to the chlorophyll *a* concentration in the leaf areas expressing pea SGRL (Fig. 5c). The maintenance of photosynthetic efficiencies in the SGRL assays reported here suggests that some of this chlorophyllide may be re-cycled into chlorophylls (Fig. 4b). Certainly further steps of the degradation pathway leading to accumulation of toxic intermediates have never been observed in assays of *SGRL*.

The data discussed above suggested a role for SGRL in general ‘housekeeping’ functions related to chlorophyll turnover during normal growth and development. A genetic screen of a diverse set of *Pisum* accessions furthermore suggested that the gene is highly conserved, with no amino acids differences detected among 19 lines (Supplementary Fig. S3a), suggesting that its role in plant development is critical. Therefore a phenotype was expected in the *sgrL*^*3421*^ (W197^STOP^) mutant plants, where the mutant SGRL protein was shown to lack activity following transient expression (Fig. 7a). The phenotypic differences observed for mutant and wild-type plants (Figs. 8, 9) are consistent with a critical, but non-essential, physiological role for SGRL in maintaining photosynthetic efficiency as a result of more efficient chlorophyll turnover. It was shown that, under conditions of high light intensity, *sgrL*^*3421*^ plants had lower photosynthetic efficiencies than those of the *cv.* Cameor control, whilst in lower light intensities their Fv/Fm values were more similar (Fig. 8). These data strongly support the idea that SGRL is important in maintaining photosynthetic efficiencies and in so doing aids normal plant development (Fig. 9). In independent experiments (Figs. 8, 9, Supplementary Fig. S10), plant growth and yield were both compromised in the mutant compared with wild-type plants, even under well-watered conditions. The expression pattern of *Arabidopsis thalianaSGRL* showed a reduction in dark incubation (Sakuraba et al. 2014b), further supporting the hypothesis that SGRL function is important in the management of photosystem turnover as a defence against high light intensities. In contrast, *sgrl*-*1* plants of *Arabidopsis thaliana* grew normally without displaying growth defects (Sakuraba et al. 2014a). It is likely that these *sgrl*-*1* mutants were not challenged sufficiently under the growth conditions used. However, direct comparisons of metabolic process between these two species are difficult, especially given that pea appears to have two SGR proteins (SGR and SGRL), whereas *Arabidopsis thaliana* has three (SGR1, SGR2 and SGRL). Although *Arabidopsis thaliana* SGRL is most similar to pea SGRL, the latter along with *Medicago trunactula* (4.0v1-3g088795.1) represent distinct SGR proteins with predicted transmembrane domains, further limiting the direct comparisons that can be made with other species.

In order to define regions of the SGRL protein responsible for activity, truncated constructs were designed to mimic the carboxy-terminal region of SGRL proteins in other species (S241^STOP^) and to remove the entire predicted transmembrane domain (M229^STOP^). The data obtained for these (Fig. 7b, c) suggested that the predicted transmembrane region was not necessary for function but its loss led to a reduction in activity. Furthermore, the SGRL^3421(M2)^ construct, encoding a transmembrane domain but lacking 40 internal amino acids, maintained some activity (Fig. 7a). These data permit the identification of two unique areas, M1-G163 and E205-P228, which are present in all forms of SGRL that showed activity in transient expression (Supplementary Fig. S8). The M1-G163 region includes the part of the SGRL protein most like SGR (Fig. 1). If both SGR and SGRL are implicated in LHCII dismantling (Rong et al. 2013; Sakuraba et al. 2014a; Horie et al. 2009; Kasuba et al. 2007), it could be concluded that the more highly conserved region is, in part, responsible for triggering the dismantling of LHCII. It may be further hypothesised that both SGR and SGRL can act as the unknown ‘factor x’ (Sato et al. 2009) at different stages of development. The much less well-conserved carboxy-terminal domains of SGR and SGRL may then be implicated in channelling chlorophyll into re-cycling or degradation pathways by either interaction with or recruitment of other proteins.

The work described in this present paper provides evidence to support the mechanism(s) discussed by exploiting induced and engineered mutants of SGRL and demonstrating similar but distinct actions for SGR and SGRL. Furthermore, the studies of the TILLING mutants support the importance of the SGRL protein, during normal and stress growth conditions. Further studies will be needed to clarify the unique contribution that SGRL proteins with transmembrane domains, as identified for pea and *Medicagotruncatula*, may make to chlorophyll turnover in plants, compared with plant species which appear to lack these proteins. Understanding how chlorophyll metabolism may be coordinated during growth and development provides knowledge that will impact on plant productivity and food security.

## Electronic supplementary material

Supplementary Fig. S1 **a** Classes of genes identified from the library representing drought-responsive genes in leaves of pea (*Pisum sativum* L.), categorised according to predicted function. **b** induced dehydrin transcripts (upper panel) in RNA from drought-stressed plants (three samples, right), compared with RNA from well-watered plants (three samples, left) and compared with expression of a control gene (His-Asp phosphorelay) in the same RNA samples (lower panel) (TIFF 83 kb)

Supplementary Fig. S2 Transmembrane domain predictions using TMHMM for: **a** Ps-SGRL discussed in this paper. **b** Medicago-4.0v. **c** Gm-LOC100792871. **d** Ps-SGR, where vertical red lines indicate presence of a predicted transmembrane domain (TIFF 52 kb)

Supplementary Fig. S3 **a** Genomic alignment displaying sequence diversity within *SGRL* of *Pisum*. Exons are shown in italics and bold; start and stop codons, SNPs, indels and the earliest start of transcription in *cv.* Cameor are colour-coded, as indicated underneath. Sequence identity is displayed by * underneath the alignment **b**: Allele-specific PCR amplification of *SGRL* ‘types’ within *Pisum*, showing the five classes distinguished based on intronic variation; from 19 genotypes tested (see Methods), a subset of lines were amplified with each diagnostic primer pair (Supplementary Table). Each panel (from left to right, three top and three lower panels) consists of Cameor and Princess (Cameor type); JI 2202; JI 281; JI 15; JI 1194 and JI 1201; and negative (no DNA) control. One class is distinguished by non-restriction with Bam HI (JI 1201 type; two lines). Exonic primers show invariant products in all tracks (SGRL control panel). The position of DNA markers is shown in the middle panels (top right, bottom left) **c**: Comparison of *SGRL* promoter sequences of *cv.* Cameor and JI 2822, showing the sequences immediately upstream of the ATG, which include the determined 5’ UTR. Differences in promoter motifs and insertions/deletions and start of transcription are highlighted; the former are predicted according to PLANT CARE (http://bioinformatics.psb.ugent.be/webtools/plantcare/html/). Sequence identity is displayed by * underneath the alignment (PDF 141 kb)

Supplementary Fig. S4 Map position of *SGRL* (red box) on pea LG III, as determined by CAPS marker analysis of recombinant inbred lines derived from JI 281 x JI 399. The positions of a number of other genes are shown, including the closely linked *Adh1* (PDF 164 kb)

Supplementary Fig. S5 **a** Phenotype of leaves of *Nicotiana benthamiana* showing effects of transiently expressed gene constructs; *SGR*, *PaO*, *SGR* plus *PaO* at 72 h post-infiltration (left) and Empty (Empty Vector), *SGR*, *SGR* plus *PaO*, *PaO* at 144 h post-infiltration (right). **b** Chlorophyll (*a*, *b* and total) concentration of leaf areas following transient expression for 144 h (see **a**). **c** Fv/Fm values determined, following infiltration of *PaO*, *SGR* and their combination, at 48 h post-infiltration (N.S = Non-Significant, *** = p < 0.001). **d** Non-denaturing protein analysis of leaf areas transiently expressing *PaO* and Empty (Empty Vector) (120 h after infiltration); total proteins are shown on the left, stained blue, and Western Blot analysis for His-tagged *PaO* is shown on the right; to the left of each panel pre-stained SeeBlue Plus2 (Invitrogen) marker is shown for reference. **e** Western blots of two-dimensional analysis of leaf areas transiently expressing *PaO* (upper) and Empty (Empty Vector; lower) (144 h after infiltration); to the left of each panel pre-stained SeeBlue Plus2 (Invitrogen) marker is shown with sizes indicated (kDa x10^-3^). The position of His-tagged PaO is indicated by a star in **d** and **e** (TIFF 209 kb)

Supplementary Fig. S6 Supporting data for **Fig. 6b** showing that the effect of co-infiltrating an unrelated gene (*GFP*) has no impact upon the activity of genes involved in the chlorophyll degradation pathway. A summary of pairwise significance values is presented below for both 48 h (left) and 72 h (right) after infiltration, with yellow highlighted boxes indicating comparisons that showed no significant differences (N.S = Non-Significant, * = p < 0.05, ** = p < 0.01, *** = p < 0.001) (TIFF 46 kb)

Supplementary Fig. S7 Phenotype of leaves showing effects of transiently expressed constructs; Empty (Empty Vector), *SGR* (OD_600_ 0.03), *SGRL* and its derivative constructs S241 (S241^STOP^), M229 (M229^STOP^), and combinations of *SGRL*, S241 (S241^STOP^) and M229 (M229^STOP^) with *SGR* (OD_600_ 0.03) at 72 h (top) and 144 h (bottom) post-infiltration (TIFF 266 kb)

Supplementary Fig. S8 Sequence of pea SGRL (upper line) showing the predicted transmembrane domain (highlighted yellow); underneath are the protein regions which are common to all SGRL constructs that display activity in transient expression assays. The dashed line indicates the extent of deletions in constructs that did not abolish activity. The positions of early stop codons, reflecting that of a TILLING mutant (W197^STOP^) and two engineered mutants (S241^STOP^) and (M229^STOP^), are shown (red) (PDF 38 kb)

Supplementary Fig. S9 Phenotype of leaves showing effects of transiently expressed *SGRL* mutant constructs; Empty (Empty Vector), 3421 (W197^STOP^) 3421(M2), *SGRL* (left) and 3421(W197^STOP^), S241 (S241^STOP^), *SGRL* and M229 (M229^STOP^) (right) at 144 h post-infiltration (TIFF 79 kb)

Supplementary Fig. S10 **a** Phenotype of *cv.* Cameor (left) and *sgrL* ^*3421*^ (right) plants grown in well-watered (top) or drought-stressed (bottom) conditions. **b** Mean plant height (cm) of *cv.* Cameor and *sgrL* ^*3421*^ mutant under well-watered or drought-stress conditions with significance values determined by t-tests of data for *cv.* Cameor and mutant plants (*** = p < 0.001) (TIFF 467 kb)

Supplementary material 11 (PDF 106 kb)
