# Supplementary figures and images for: SGRL can regulate chlorophyll metabolism and contributes to normal plant growth and development in Pisum sativum L
Source: Plant Mol Biol. 2015 Sep 7;89(6):539–58. doi: 10.1007/s11103-015-0372-4 (PMC4659853; doi:10.1007/s11103-015-0372-4)

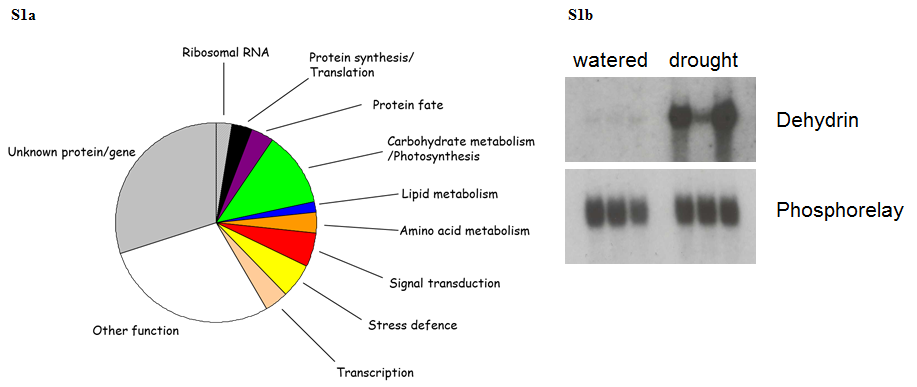

Supplement: Supplementary file 1 — a Classes of genes identified from the library representing drought-responsive genes in leaves of pea (Pisum sativum L.), categorised according to predicted function. b induced dehydrin transcripts (upper panel) in RNA from drought-stressed plants (three samples, right), compared with RNA from well-watered plants (three samples, left) and compared with expression of a control gene (His-Asp phosphorelay) in the same RNA samples (lower panel) (TIFF 83 kb) [file 11103_2015_372_MOESM1_ESM.tif]

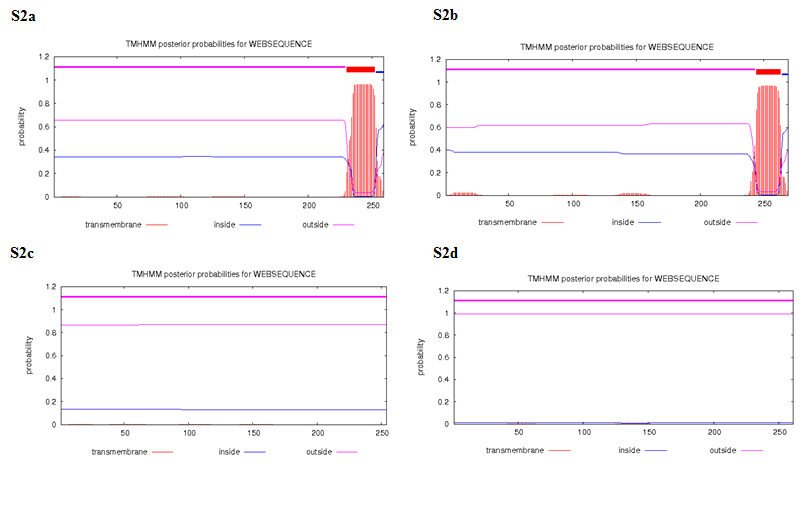

Supplement: Supplementary file 2 — Transmembrane domain predictions using TMHMM for: a Ps-SGRL discussed in this paper. b Medicago-4.0v. c Gm-LOC100792871. d Ps-SGR, where vertical red lines indicate presence of a predicted transmembrane domain (TIFF 52 kb) [file 11103_2015_372_MOESM2_ESM.tif]

S4

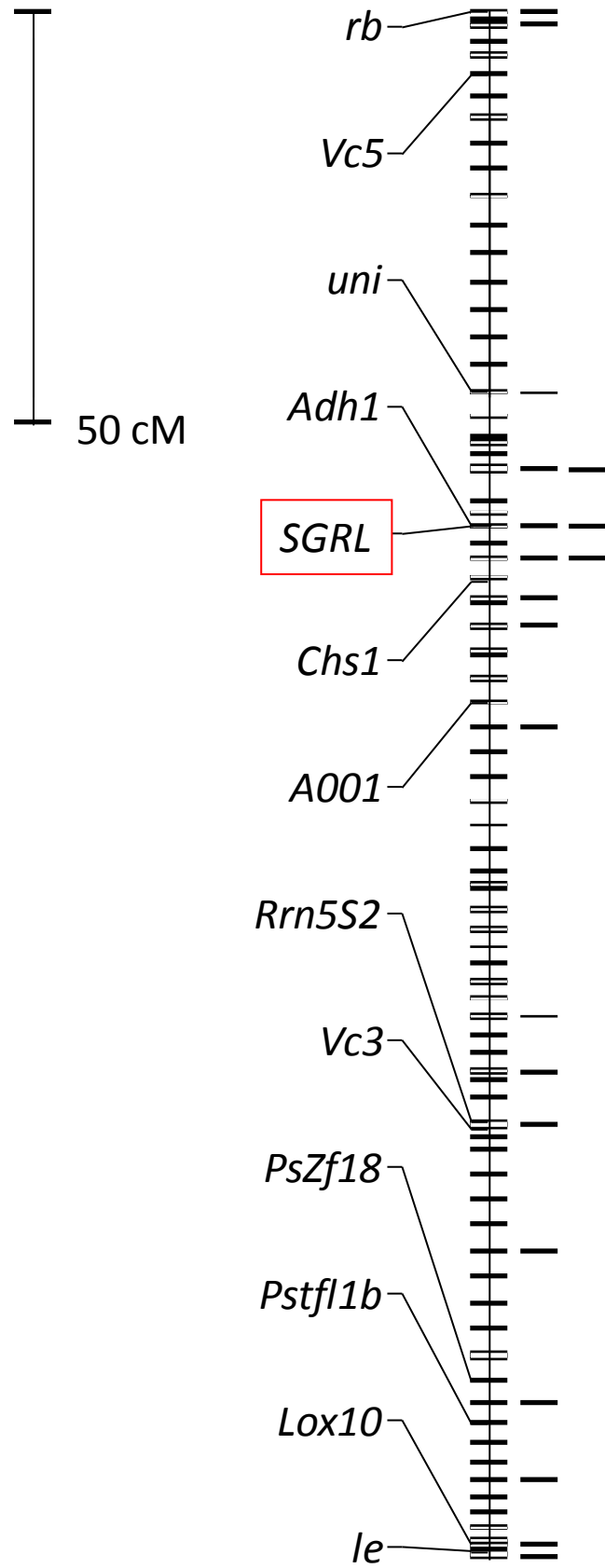

Supplement: Supplementary file 4 — Map position of SGRL (red box) on pea LG III, as determined by CAPS marker analysis of recombinant inbred lines derived from JI 281 x JI 399. The positions of a number of other genes are shown, including the closely linked Adh1 (PDF 164 kb) [file 11103_2015_372_MOESM4_ESM.pdf]

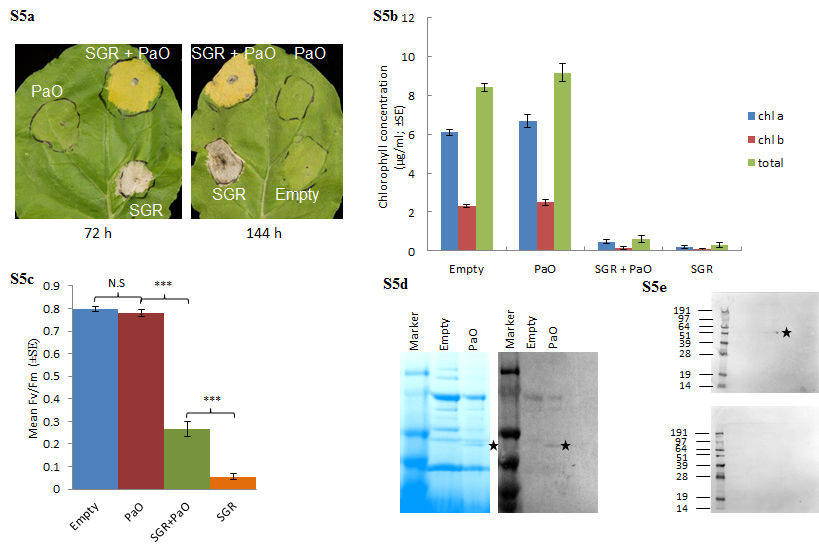

Supplement: Supplementary file 5 — a Phenotype of leaves of Nicotiana benthamiana showing effects of transiently expressed gene constructs; SGR, PaO, SGR plus PaO at 72 h post-infiltration (left) and Empty (Empty Vector), SGR, SGR plus PaO, PaO at 144 h post-infiltration (right). b Chlorophyll (a, b and total) concentration of leaf areas following transient expression for 144 h (see a). c Fv/Fm values determined, following infiltration of PaO, SGR and their combination, at 48 h post-infiltration (N.S = Non-Significant, *** = p < 0.001). d Non-denaturing protein analysis of leaf areas transiently expressing PaO and Empty (Empty Vector) (120 h after infiltration); total proteins are shown on the left, stained blue, and Western Blot analysis for His-tagged PaO is shown on the right; to the left of each panel pre-stained SeeBlue Plus2 (Invitrogen) marker is shown for reference. e Western blots of two-dimensional analysis of leaf areas transiently expressing PaO (upper) and Empty (Empty Vector; lower) (144 h after infiltration); to the left of each panel pre-stained SeeBlue Plus2 (Invitrogen) marker is shown with sizes indicated (kDa x10-3). The position of His-tagged PaO is indicated by a star in d and e (TIFF 209 kb) [file 11103_2015_372_MOESM5_ESM.tif]

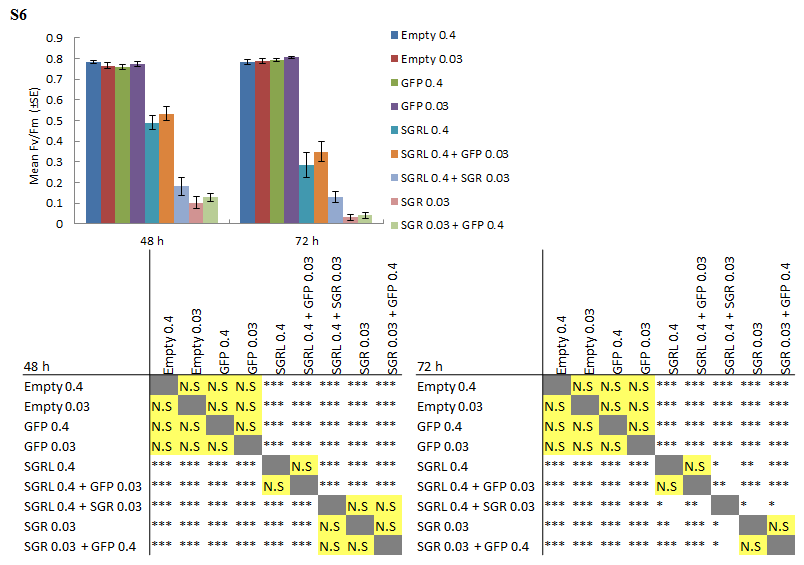

Supplement: Supplementary file 6 — Supporting data for Fig. 6b showing that the effect of co-infiltrating an unrelated gene (GFP) has no impact upon the activity of genes involved in the chlorophyll degradation pathway. A summary of pairwise significance values is presented below for both 48 h (left) and 72 h (right) after infiltration, with yellow highlighted boxes indicating comparisons that showed no significant differences (N.S = Non-Significant, * = p < 0.05, ** = p < 0.01, *** = p < 0.001) (TIFF 46 kb) [file 11103_2015_372_MOESM6_ESM.tif]

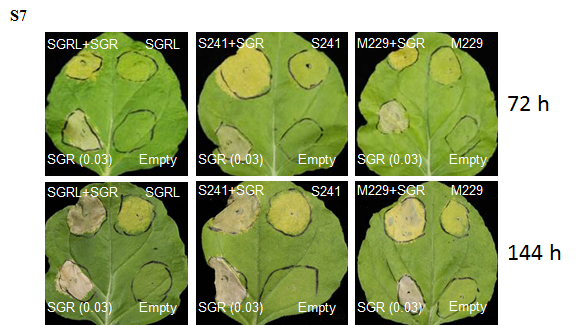

Supplement: Supplementary file 7 — Phenotype of leaves showing effects of transiently expressed constructs; Empty (Empty Vector), SGR (OD600 0.03), SGRL and its derivative constructs S241 (S241STOP), M229 (M229STOP), and combinations of SGRL, S241 (S241STOP) and M229 (M229STOP) with SGR (OD600 0.03) at 72 h (top) and 144 h (bottom) post-infiltration (TIFF 266 kb) [file 11103_2015_372_MOESM7_ESM.tif]

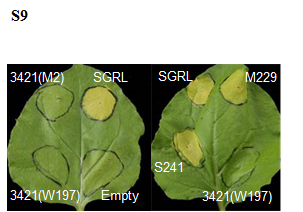

Supplement: Supplementary file 9 — Phenotype of leaves showing effects of transiently expressed SGRL mutant constructs; Empty (Empty Vector), 3421 (W197STOP) 3421(M2), SGRL (left) and 3421(W197STOP), S241 (S241STOP), SGRL and M229 (M229STOP) (right) at 144 h post-infiltration (TIFF 79 kb) [file 11103_2015_372_MOESM9_ESM.tif]

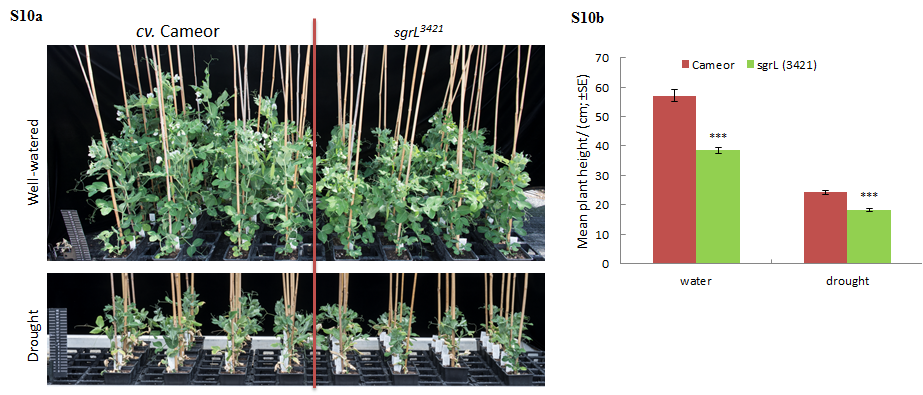

Supplement: Supplementary file 10 — a Phenotype of cv. Cameor (left) and sgrL 3421 (right) plants grown in well-watered (top) or drought-stressed (bottom) conditions. b Mean plant height (cm) of cv. Cameor and sgrL 3421 mutant under well-watered or drought-stress conditions with significance values determined by t-tests of data for cv. Cameor and mutant plants (*** = p < 0.001) (TIFF 467 kb) [file 11103_2015_372_MOESM10_ESM.tif]
