# Supplementary material for: SGRL can regulate chlorophyll metabolism and contributes to normal plant growth and development in Pisum sativum L
Source: Plant Mol Biol. 2015 Sep 7;89(6):539–58. doi: 10.1007/s11103-015-0372-4 (PMC4659853; doi:10.1007/s11103-015-0372-4)
Supplement: Supplementary file 3 — a Genomic alignment displaying sequence diversity within SGRL of Pisum. Exons are shown in italics and bold; start and stop codons, SNPs, indels and the earliest start of transcription in cv. Cameor are colour-coded, as indicated underneath. Sequence identity is displayed by * underneath the alignment b: Allele-specific PCR amplification of SGRL ‘types’ within Pisum, showing the five classes distinguished based on intronic variation; from 19 genotypes tested (see Methods), a subset of lines were amplified with each diagnostic primer pair (Supplementary Table). Each panel (from left to right, three top and three lower panels) consists of Cameor and Princess (Cameor type); JI 2202; JI 281; JI 15; JI 1194 and JI 1201; and negative (no DNA) control. One class is distinguished by non-restriction with Bam HI (JI 1201 type; two lines). Exonic primers show invariant products in all tracks (SGRL control panel). The position of DNA markers is shown in the middle panels (top right, bottom left) c: Comparison of SGRL promoter sequences of cv. Cameor and JI 2822, showing the sequences immediately upstream of the ATG, which include the determined 5’ UTR. Differences in promoter motifs and insertions/deletions and start of transcription are highlighted; the former are predicted according to PLANT CARE (http://bioinformatics.psb.ugent.be/webtools/plantcare/html/). Sequence identity is displayed by * underneath the alignment (PDF 141 kb) [file 11103_2015_372_MOESM3_ESM.pdf]

S3a

```
JI1201  ------------------------------------------------------------
JI281   ----------------------------------------------------------AC 2
JI2202  ------------------------------------------------------------
Cameor  GATTTTGGATTGTTGCGGTGTTTGAACAGAATCACACAGTCCACACGAACAAACCAAACT 60
JI2822  GATTTTGGATTGTTGCGGTGTTTGAACAGAATCACACAGTCCACAAGAACAAACCAAACT 60

JI1201  ---------------------------------------------------------CTT 3
JI281   TGAGACACTAATTTCCCACATCACTCCTTCTTTCTCTCTCTTT--CTATCTCTAACTCTT 60
JI2202  ----------ATTTCCCACATCACTCTTTCTTTCTCTCTCTTTTTCTATCTCTAACTCTT 50
Cameor  GAGAACACTAATTTCCCACATCACTCCTTCTTTCTCTCTCTTT--CTATCTCTAACTCTT 118
JI2822  GAGAACACTAATTTCCCACATCACTCTTTCTTTCTCTCTCTTT--CTATCT-TAACTCTT 117
                                                                 ***

JI1201  TTAACTACAGAAGAACAAGGAAAGAAGATAATGGTGGTGACATGTGAAACATGAAGAGGA 63
JI281   TTAACAGCAGAAGAACAAGGAAAGAAGATAATGGTGGTGACATGTGAAACATGAAGAGTA 120
JI2202  TTAACTACAGAAGAACAAGGAAAGAAGATAATGGTTCTGACATGTGAAACATGAAGAGGA 110
Cameor  TTAACAGCAGAAGAACAAGGAAAGAAGATAATGGTGGTGACATGTGAAACATGAAGAGTA 178
JI2822  TTAACTACAGAAGAACAAGGAAAGAAGATAATGGTGGTGACATGTGAAACATGAAGAGGA 177
        *****  ***************************  ********************* *

JI1201  CTGTATTATTATTATGCATCTTCAATGGCATCATTATGTCATAATGCCTTTTCATTTTCA 123
JI281   CTGTATTATTAT---GCATCTTCAATGGCATCATTATGTCATAATGCCTTTTCATTTTCA 177
JI2202  CTGTATTATTAT---GCATCTTCAATGGCATCATTATGTCATAATGCCTTTTCATTTTCA 167
Cameor  CTGTATTATTAT---GCATCTTCAATGGCATCATTATGTCATAATGCCTTTTCATTTTCA 235
JI2822  CTGTATTATTATTATGCATCTTCAATGGCATCATTATGTCATAATGCCTTTTCATTTTCA 237
        ************   *********************************************

JI1201  CCTACAAAACTTTTCCCTATCATGTTAAAGCCATCTTTCAGATGCTCCTCCATTACTACT 183
JI281   CCTACAAAACTTTTCCCTATCATGTTAAAGCCATCTTTCAGATGCTCCTCCATTACTACT 237
JI2202  CCTACAAAACTTTTCCCTATCATGTTAAAGCCATCTTTCAGATGCTCCTCCATTACTACT 227
Cameor  CCTACAAAACTTTTCCCTATCATGTTAAAGCCATCTTTCAGATGCTCCTCCATTACTACT 295
JI2822  CCTACAAAACTTTTCCCTATCATGTTAAAGCCATCTTTCAGATGCTCCTCCATTACTACT 297
        ************************************************************

JI1201  AATTCCACACCTTCCTACAATTCCATTGTTTTTGAGGTTCCTTTAACTCTCTTTCTCTCT 243
JI281   AATTCCACACCTTCCTACAATTCCATTGTTTTTGAGGTTCCTTTAACTCTCT----CTCT 293
JI2202  AATTCCACACCTTCCTACAATTCCATTGTTTTTGAGGTTCCTTTAACTCTCTTTCTCTCT 287
Cameor  AATTCCACACCTTCCTACAATTCCATTGTTTTTGAGGTTCCTTTAACTCTCT----CTCT 351
JI2822  AATTCCACACCTTCCTACAATTCCATTGTTTTTGAGGTTCCTTTAACTCTCT----CTCT 353
        ****************************************************    ****

JI1201  TTCACTTTCTTATTATGCATTCAATATTAATACTTAATGTAGCAATTTGCAATCTTTATA 303
JI281   TTCACTTTCTTATTATGCATTCAATATTAATACTTAATGTAGCAATTTGCAATCTTTATA 353
JI2202  TTCACT----TATTATGCATTCAATATTAATACTTAATGTAGCAAGTTGCAATCTTTATA 343
Cameor  TTCACTTTCTTATTATGCATTCAATATTAATACTTAATGTAGCAAGTTGCAATCTTTATA 411
JI2822  TTCACTTTCTTATTATGCATTCAATATTAATACTTAATGTAGCAAGTTGCAATCTCTATA 413
        ******    *********************************** ********* ****

JI1201  CCTATGCATGAGAAACCTCATTACTCATATGTCAATGTGTAGAATGAATTGTGCTTCTTC 363
JI281   CCTATGCATGAGAAACCTCATTACTCATATGTCAATGTGTAGAATGAATTGTGCTTCTTC 413
JI2202  TCTATGCATGAGAAACCTCAATACTCATATGTCAATGTGTAGAATGAATTGTGCTTCTTC 403
Cameor  CCTATGCATGAGAAACCTCATTACTCATATGTCAATGTGTAGAATGAATTGTGCTTCTTC 471
JI2822  CCTATGCATGAGAAACCTCATTACTCATATGTCAATGTGTAGAATGAATTGTGCTTCTTC 473
         ******************* ***************************************

JI1201  TATATTACTTCATGATCCGTGGATTTGTTAAACATTTTCACTTTTCTTGATTTGTTATGA 423
JI281   TATATTACTTCGTGATCCGTGGATTTGTTAAACATTTTCACTTTTCTTGATTTGTTATGA 473
JI2202  TATATTACTTCATGATCCGTGGATTTGTTAAACATTTTCACTTTTCTTGATTTGTTATGA 463
Cameor  TATATTACTTCATGATCCGTGGATTTGTTAAACATTTTCACTTTTCTTCATTTGTTATGA 531
JI2822  TATATTACTTCATGATCCGTGGATTTGTTAAACATTTTCACTTTTCTTGATTTGTTATGA 533
        *********** ************************************ ***********
```

```
JI1201 CTGCTTTGATTCATTCAAGTTATCACTGAATTAGGGTTTAGGGATTAAACTGTTTACAAA 483
JI281  CTGCTTTGATTCATTCAAGTTATCACTGAATTAGGGTTTAGGGATTAAACTGTTTACAAA 533
JI2202 CTGCTTTGATTCATTCAAGTTATCACTGAATTAGGGTTTAGGGATTAAACTGTTTACAAA 523
Cameor CTGCTTTGATTCATTCAAGTTATCACTGAATTAGGGTTTAGGGATTAAACTGTTTACAAA 591
JI2822 CTGCTTTGATTCATTCAAGTTATCACTGAATTAGGGTTTAGGGATTAAACTGTTTACAAA 593
       ************************************************************

JI1201 ATTATGATTCATGAGATAGTTCCTGCAATTGAAAATATGCCTATAGGAATACGAATCCAT 543
JI281  ATTATGATTCATGAGATAGTTCCTGCAATTGAAAATATGCCTATAGGAATACGAATCCAT 593
JI2202 ATTATGATTCATGAGATAGTTCCTGCAATTGAAAATATGCCTATAGGAATACGAATCCAT 583
Cameor ATTATGATTCATGAGATAGTTCCTGCAATTGAAAATATGCCTATAGGAATACGAATCCAT 651
JI2822 ATTATGATTCATGAGATAGTTCCTGCAATTGAAAATATGCCTATAGGAATACGAATCCAT 653
       ************************************************************

JI1201 GATTAACCATAGTAGTCTATAATTGTTATTTAGATGAAGAAAACAGTGAATTTCTTCTGT 603
JI281  GATTAACCATAGTAGTCTATAATTGTTATTTGGATGAAGAAAACAGTGAATTTCTTCT-- 651
JI2202 GATTAACCATAGTAGTCTATAATTGTTATTTGGATGAAGAAAACAGTGAATTTCTTCTGT 643
Cameor GATTAACCATAGTAGTCTATAATTGTTATTTAGATGAAGAAAACAGTGAATTTCTTCTGT 711
JI2822 GATTAACCATACTAGTCTATAATTGTTATTTAGATGAAGAAAACAGTGAATTTCTTCTGT 713
       *********** ******************* **************************  

JI1201 CTCTTGAATATCATTTAGGATCTTATTTATATTCATTATATAAGATTTTTACCTGTTTTT 663
JI281  --------------------------TTATATTCATTATGTAAGATTT--ACCTGTTTTT 683
JI2202 CTCTTGAATATTATTTAGGATCTTATTTATATTCATTATATAAGATTT--ACCTGTTTTT 701
Cameor CTCTTGAATATTATTTAGGATCTTATTTATATTCATTATATAAGATTTTTACCTGTTTTT 771
JI2822 CTCTTGAATATTATTTAGGATCTTATTTATATTCATCATATAAGATTT--ACCTGTTTTT 771
                                 ********** ** ********  **********

JI1201 TGTTTTTTTTTTGTTTTTTATCTTAGACTGTTAGGCTATTGGGTCCTCCAACAAAATTTG 723
JI281  TGTTTTTTTTTTGTTTTTTATCTTAGACTGTTAGGCTATTGGGTCCTCCAACAAAATTTG 743
JI2202 TGTTTTTTTTT-GTTTTTAATCTTAGACTGTTAGGCTATTGGGTCCTCCAACAAAATTTG 760
Cameor TGTTTTTTTTTTGTTTTTTATCTTAGACTGTTAGGCTATTGGGTCCTCCAACAAAATTTG 831
JI2822 TGTTTTTTTTTTTTTTTTTATCTTAGACTGTTAGGCTATTGGGTCCTCCAACAAAATTTG 831
       ***********  ***** *****************************************

JI1201 AAGCTTCAAAGCTGAAGGTTGTTCTATTGGAAGATCAGATTAACAGATATGCAAGTATTA 783
JI281  AAGCTTCAAAGCTGAAGGTTGTTCTATTGGAAGATCAGATTAACAGATATGCAAGTATTA 803
JI2202 AAGCTTCAAAGCTGAAGGTTGTTCTATTGGAAGATCAGATAAACAGATATGCAAGTATTA 820
Cameor AAGCTTCAAAGCTGAAGGTTGTTCTATTGGAAGATCAGATTAACAGATATGCAAGTATTA 891
JI2822 AAGCTTCAAAGCTGAAGGTTGTTCTATTGGAAGATCAGATTAACAGATATGCAAGTATTA 891
       **************************************** *******************

JI1201 TCCCAAGAACCTACATTCTATCCCACTGTGATTTAACAGCTAATCTCACTTTAGCTGTTT 843
JI281  TTCCAAGAACCTACATTCTATCCCACTGCGATTTAACAGCTAATCTCACTTTAGCTGTTT 863
JI2202 TCCCAAGAACCTACATTCTATCCCACTGTGATTTAACAGCTAATCTCACTTTAGCTGTTT 880
Cameor TCCCAAGAACCTACATTCTATCCCACTGTGATTTAACAGCTAATCTCACTTTAGCTGTTT 951
JI2822 TCCCAAGAACCTACATTCTATCCCACTGTGATTTAACAGCTAATCTCACTTTAGCTGTTT 951
       * ************************** *******************************

JI1201 CCAATGTCATCAAACTCGAGCAGGTTTGTGTCTTTATGTTTTGCCTGCTTTGGCGTGGAA 903
JI281  CCAATGTCATCAAACTCGAGCAGGTTTGTGTCTTTATGTTTTGCCTGCTTTGGCGTGGAA 923
JI2202 CCAATGTCATCAAACTCGAGCAGGTTTGTGTCTTTATGTTTTGCCTGCTTTGGCGTGGAA 940
Cameor CCAATGTCATCAAACTCGAGCAGGTTTGTGTCTTTATGTTTTGCCTGCTTTGGCGTGGAA 1011
JI2822 CCAATGTCATCAAACTCGAGCAGGTTTGTGTCTTTATGTTTTGCCTGCTTTTGCGTGGAA 1011
       *************************************************** ********

JI1201 AATTAGTCTTTGACGCATATTTTGAACGAAGGAAAATGCTAACAAGTGCCTTTAGAAAAT 963
JI281  AATTAGTCTTTGACGCATATTTTGAACGAAGGAAAATGCTAACAAGTGCCTTTAGAAAAT 983
JI2202 AATTAGTCTTTGACGCATATTTTGAACGAAGGAAAATGCTAACAAGTGCCTTTAGAAAAT 1000
Cameor AATTAGTCTTTGACGCATATTTTGAACGAAGGAAAATGCTAACAAGTGCCTTTAGAAAAT 1071
JI2822 AATTAGTCTTTGACGCATCTTTTTAACGAAGGAAAATGCTAACATGTGCCTTTAGAAAAT 1071
       ****************** **** ******************** ***************
```

```
JI1201 TGAATGCGTTT-TTGCAAAATACACAATTCCTTTGATACTTCTTTTAAGTAATTGAACCC 1022
JI281  TGAATGCGTTTATTGCAAAATACACAATTCCTTTGATACTTCTTTTAAGTAATTGAACGC 1043
JI2202 TGAATGCGTTTATTGCAAAATACACAATTCCTTTGATACTTCTTTTAAGTAATTGAACGC 1060
Cameor TGAATGCGTTT-TTGCAAAATACACAATTCCTTTGATACTTCTTTTAAGTAATTGAACCC 1130
JI2822 TGAACGCATTTATTGCAAAATACACAATTCGTTTGATACTTCTTTTAAGTG--------- 1122
       **** ** *** ****************** *******************

JI1201 ATTTTTTAGGGTGCCCCTTAGGTTGTTCATTAGTATAAGTTATTGCTTAATTAGCACATG 1082
JI281  ATTTTTTAGGGTGCCCCTTAGGTTGTTCATTAGTATAAGTTATTGCTTAATTAGCACATG 1103
JI2202 ATTTTTTAGGGTGCCCCTTAGGTTGTTCATTAGTATA------TGCTTAATTAGCACATG 1114
Cameor ATTTTTTAGGGTGCCCCTTAGGTTGTTCATTAGTATAAGTTATTGCTTAATTAGCACATG 1190
JI2822 --------GGGTTCCCCTTAGGTTGTTCATTAGTATAAGTTATTGCTTAATTAGCACATG 1174
               **** ************************      *****************

JI1201 TTTGATGTCTTGTATTCCTTGTGTGAAATGGCAGTTGAGAGGGTGGTACCAGAAGGATGA 1142
JI281  TTTGATGTCTTGTATTCCTTGTGTGAAATGGCAGTTGAGAGGGTGGTACCAGAAGGATGA 1163
JI2202 TTTGATGTCTTGTATTCCTTGTGTGAAATGGCAGTTGAGAGGGTGGTACCAGAAGGATGA 1174
Cameor TTTGATGTCTTGTATTCCTTGTGTGAAATGGCAGTTGAGAGGGTGGTACCAGAAGGATGA 1250
JI2822 TTTGATGTCTTGTATTCCTTGTGTGAAATGGCAGTTGAGAGGGTGGTACCAGAAGGATGA 1234
       ************************************************************

JI1201 TGTTGTAGCTGAATGGAAGAAAGTGAAGAATGAAATGTGCCTACATGTTCATTGCTTTGT 1202
JI281  TGTTGTAGCTGAATGGAAGAAAGTGAAGAATGAAATGTGCCTACATGTTCATTGCTTTGT 1223
JI2202 TGTTGTAGCTGAATGGAAGAAAGTGAAGAATGAAATGTGCCTACATGTTCATTGCTTTGT 1234
Cameor TGTTGTAGCTGAATGGAAGAAAGTGAAGAATGAAATGTGCCTACATGTTCATTGCTTTGT 1310
JI2822 TGTTGTAGCTGAATGGAAGAAAGTGAAGAATGAAATGTGCCTACATGTTCATTGCTTTGT 1294
       ************************************************************

JI1201 TAGTGGTCCTAATTCCTTCCTAGATCTCGCTGCTGAGTTTAGATATCACATTTTCTCCAA 1262
JI281  TAGTGGTCCTAATTCCTTCCTAGATCTCGCTGCTGAGTTTAGATATCACATTTTCTCCAA 1283
JI2202 TAGTGGTCCTAATTCCTTCCTAGATCTCGCTGCTGAGTTTAGATATCACATTTTCTCCAA 1294
Cameor TAGTGGTCCTAATTCCTTCCTAGATCTCGCTGCTGAGTTTAGATATCACATTTTCTCCAA 1370
JI2822 TAGTGGTCCTAATTCCTTCCTAGATCTCGCTGCTGAGTTTAGATATCACATTTTCTCCAA 1354
       ************************************************************

JI1201 GGAAATGCCTTTGGTATGTTTGCGTTTAATTTATATTATCCCCTTAATGCATCCAATTCC 1322
JI281  GGAAATGCCTTTGGTATGTTTGCGTTTAATTTATATTATCCCCTTAATGCATCCAATTCC 1343
JI2202 GGAAATGCCTTTGGTATGTTTGAGTTTAATTTACATTATCCCCTTAATGCATCCAATTCC 1354
Cameor GGAAATGCCTTTGGTATGTTTGCGTTTAATTTATATTATCCCCTTAATGCATCCAATTCC 1430
JI2822 GGAAATGCCTTTGGTATGTTTGCGTTTAATTTATATTATCCCCTTAATGCATCCAATTCC 1414
       ********************** ********** **************************

JI1201 TATCAAAACAGGAATAATCTGAGAACTAGAACTAGGACTGTTCTAACATGTTCTCCCTCT 1382
JI281  TATCAAAACAGGAATAATCTGAGAACTAGAACTAAGACTGTTCTAACGTGTTCTCCCTCT 1403
JI2202 TATCAAAACAGGAATAATCTGAGAACTAGAACTAGGACTGTTCTAACGTGGTCTCCCTCT 1414
Cameor TATCAAAACAGGAATAATCTGAGAACTAGAACTAGGACTGTTCTAACATGTTCTCCCTCT 1490
JI2822 TATCAAAACAGGAATAATCTGAGAACTAGAACTAGGACTGTTCTAACGTGTTCTCTCTCT 1474
       ********************************** ************ ** **** ****

JI1201 GGACTCAAATATAAGCAAAAAATAGTCATATGCGCACCAATTAAAAATTTCAGTGAAATT 1442
JI281  GGACTCAAATATAAGCAAAAAATAGTCATATGCGCACCAATTAAAAATGTCAGTGAAATT 1463
JI2202 GGACTCAAATATAAGCAAAAAATAGTCATATGCGCACCAATTAAAAATGTCAGTGAAATT 1474
Cameor GGACTCAAATATAAGCAAAAAATAGTCATATGCGCACCAATTAAAAATTTCAGTGAAATT 1550
JI2822 GGACTTAAATATAAGCAAAAAATAGTCATACACGCACCAATTAAAAATGTCAGTGAAATT 1534
       ***** ************************  **************** ***********

JI1201 TTCTTCATGAAATAGAAGATGATTGTACTCTCCAAAGTCCAAACTCCTGTTTCTCTTCAT 1502
JI281  TTCTTCATGAAATAGAAGATGATTGTACTCTCCAAAGTCCAAACTCCTGTTTCTCTTCAT 1523
JI2202 TTCTTCATGAAATAGAAGATGATTGTACTCTCCAAAGTCCAAACTCCTGTTTCTCTTCAT 1534
Cameor TTCTTCATGAAATAGAAGATGATTGTACTCTCCAAAGTCCAAACTCCTGTTTCTCTTCAT 1610
JI2822 TTCTTCATGAAATAGAAGATGATTGTATTCTCCAAAGTCCAAACTCCTGTTTCTCTTCAT 1594
       *************************** ********************************
```

```
JI1201 CTAATTTTCTATCAGAATTTGTTTGGTAACGCTAAACATGGTGAAGTTAGTTAGAAATTG 1562
JI281  CTAATTTTCCATCAGAATTTGTTTGGTAACGCTAAACATGGTGAAGTTAGTTAGAAATTG 1583
JI2202 C------------AGAATTTGTTTGGTAACGCTAAACATGGTGAAGTTAGTTAGAAATTG 1582
Cameor CTAATTTTCTATCAGAATTTGTTTGGTAACGCTAAACATGGTGAAGTTAGTTAGAAATTG 1670
JI2822 CTAATTTTCTATCAGAATTTGTTTGGTAACGCTAAACATGGTGAAGTTAGTTAGAAATTG 1654
       *            ***********************************************

JI1201 TTTATGTTTGAGTCCGGAGGTAGTATTCTACATTGCAACCTTATCGAAGAGTTGCATATG 1622
JI281  TTTATGTTTGAGTCTGGAGGTAGTATTCTACATTGCAACCTTATCGAAGAGTTGCATATG 1643
JI2202 TTTATGTTTGAGTCCGGAGGTAGTATTCTACATTGCAACCTTATCGAAGAGTTGCATATG 1642
Cameor TTTATGTTTGAGTCCGGAGGTAGTATTCTACATTGCAACCTTATCGAAGAGTTGCATATG 1730
JI2822 TTTATGTTTGAGTCCGGAGGTAGTATTCTACATTGCAACCTTATCGAAGAGTTGCATATG 1714
       ************** *********************************************

JI1201 GATCGCCAATTAGTGAACTGTTTGTTGCTAATTGCTAAAGCATGTCATTGCTCAAAATGA 1682
JI281  GATCCCCAATTAGTGAACTGTTTGTTGCTAATTGCTAAAGCATGTCATTGCTCAAAATGA 1703
JI2202 GATCCCCAATTAGTGAACTGTTTGTTGCTAATTGCTAAAGCATGTCATTGCTCAAAATGA 1702
Cameor GATCCCCAATTAGTGAACTGTTTGTTGCTAATTGCTAAAGCATGTCATTGCTCAAA---- 1786
JI2822 GATCCCCAATTAGTGAACTGTTTGTTGCTAATTGCTAAAGCATGTCATTGCTCAAAATGA 1774
       **** ***************************************************    

JI1201 ACATAACAGCTTATAAAGAAGATTCACTATAAAAATATGGGGTAAGAAACGAGAATTTAA 1742
JI281  ACATAACAGCTTATAAAGAAGATTCACTATAAAAATATGGGGTAAGAAACGAGAATTTAA 1763
JI2202 ACATAACAGCTTATAAAGAAGATTCACTATAAAAATATGGGGTAAGAAACGAGAATTTAA 1762
Cameor -------------TAAAGAAGATTCAGTATAAAAATATGGGGTAAGAAACGAGAATTTAA 1833
JI2822 ACATAACAGCTTATAAAGAAGATTCACTATAAAAATATGGGGTAAGAAACGAGAATTTAA 1834
                    ************* *********************************

JI1201 TGATAGCCTTTCGCGTAAACTTACACTTAAGCCTATTATGAACTGCAATTGTGATATACT 1802
JI281  TGATAGCCTTTCGCGTAAACTTACACTTAAGCCTATTATGAACTGCAATCGTGATATACT 1823
JI2202 TGATAGCCTTTCGCGTAAACTTACACTTAAGCCTATTATGAAGTGCAATCGTGATATACT 1822
Cameor TGATAGCCTTTCGCGTAAACTTACACTTAAGCCTATTATGAACTGCAATCGTGATATACT 1893
JI2822 TGATAGCCTTTCGCGTAAACTTACACTTAAGCCTATTATGAACTGCAATCGTGATATACT 1894
       ****************************************** ****** **********

JI1201 ATATCTGGTATGTAAATTGGTTCTCCAATTGCAGATTACTGGATTAGCAGTCCTATGCCT 1862
JI281  ATATCTGGTATGTAAATTGGTTCTCCAATTGCAGATTACTGGATTAGCAGTCCTATGCCT 1883
JI2202 ATATCTGGTATGTAAATTGGTTCTCCAATTGCAGATTACTGGATTAGCAGTCCTATGCCT 1882
Cameor ATATCTGGTATGTAAATTGGTTCTCCAATTGCAGATTACTGGATTAGCAGTCCTATGCCT 1953
JI2822 ATATCTGGTATGTAAATTGGTTCTCCAATTGCAGATTACTGGATTAGCAGTCCTATGCCT 1954
       ************************************************************

JI1201 AAATGTATTATGACACATGGTTGGATTGTTGTCTTGACCATGCCAGGTACTCAAAGCAAT 1922
JI281  AAATGTATTATGACACATGGTTGGATTGTTGTCTTGACCATGCCAGGTACTCAAAGCAAT 1943
JI2202 AAATGTATTATGACACATGGTTGGATTGTTGTCTTGACCATGCCAGGTACTCAAAGCAAT 1942
Cameor AAATGTATTATGACACATGGTTGGATTGTTGTCTTGACCATGCCAGGTACTCAAAGCAAT 2013
JI2822 AAATGTATTATGACACATGGTTGGATTGTTGTCTTGACCATGCCAGGTACTCAAAGCAAT 2014
       ************************************************************

JI1201 TCAATGCGGAGATTCTGAACTTTTCCACGAGCATCCAGAATTGCTGGATTCTATCGTTAG 1982
JI281  TCAATGCGGAGATTCTGAACTTTTCCACGAGCATCCAGAATTGCTGGATTCTATCGTTAG 2003
JI2202 TCAATGCGGAGATTCTGAACTTTTCCACGAGCATCCAGAATTGCTGGATTCTATCGTTAG 2002
Cameor TCAATGCGGAGATTCTGAACTTTTCCACGAGCATCCAGAATTGCTGGATTCTATCGTTAG 2073
JI2822 TCAATGCGGAGATTCTGAACTTTTCCACGAGCATCCAGAATTGCTGGATTCTATCGTTAG 2074
       ************************************************************

JI1201 AGTATATTTCCATTCTAGCTCAAAAATATACAACAGAATGGAATGTTGGGGGCCTTTAAG 2042
JI281  AGTATATTTCCATTCTAGCTCAAAAATATACAACAGAATGGAATGTTGGGGGCCTTTAAG 2063
JI2202 AGTATATTTCCATTCTAGCTCAAAAATATACAACAGAATGGAATGTTGGGGGCCTTTAAG 2062
Cameor AGTATATTTCCATTCTAGCTCAAAAATATACAACAGAATGGAATGTTGGGGGCCTTTAAG 2133
JI2822 AGTATATTTCCATTCTAGCTCAAAAATATACAACAGAATGGAATGTTGGGGGCCTTTAAG 2134
       ************************************************************
```

```
JI1201 GGATGCAATGGAGGTAATATTAGGAAACACTGAAATGAGTAATTTTACTATCTTTTTGAC 2102
JI281  GGATGCAATGGAGGTAATATTAGGAAACACTGAAATGAGTAATTTTACTATCTTTTTGAC 2123
JI2202 GGATGCAATGGAGGTAATATTAGGAAACACTGAAATGAGTAATTTTACTATCTTTTTGAC 2122
Cameor GGATGCAATGGAGGTAATATTAGGAAACACTGAAATGAGTAATTTTACTATCTTTTTGAC 2193
JI2822 GGATGCAATGGAGGTAATATTAGGAAACACTGAAATGAGTAATTTTACTACCTTTTTGAC 2194
       *************************************************** *********

JI1201 ATGAGTAATCTTTACTTTTGTATTTTACCTTTGCCTTTTTGTTTTGTTATCTGATTATGT 2162
JI281  ATGAGTAATCTTTACTTTTGTATTTTACCTTTGCCTTTTTGTTTTGTTATCTGATTATGT 2183
JI2202 ATGAGTAATCTTTACTTTTGTATTTTACCTTTGCCTTTTTGTTTTGTTATCTGATTATGT 2182
Cameor ATGAGTAATCTTTACTTTTGTATTTTACCTTTGCCTTTTTGTTTTGTTATCTGATTATGT 2253
JI2822 ATGAGTAATCTTTACTTTTGTATTTTACCTTTGCCTTTTTGTTTTGTTATCTGATTATGT 2254
       ************************************************************

JI1201 TTCTTCGTGTTTTCAGGGAAAACGAGGCGATCAGTTACAAGGGTTGATAAACAGAGATCG 2222
JI281  TTCTTCGTGTTTTCAGGGAAAACGAGGCGATCAGTTACAAGGGTTGATAAACAGAGATCG 2243
JI2202 TTCTTCGTGTTTTCAGGGAAAACGAGGCGATCAGTTACAAGGGTTGATAAACAGAGATCG 2242
Cameor TTCTTCGTGTTTTCAGGGAAAACGAGGCGATCAGTTACAAGGGTTGATAAACAGAGATCG 2313
JI2822 TTCTTCGTGTTTTCAGGGAAAACGAGGCGATCAGTTACAAGGGTTGATAAACAGAGATCG 2314
       ************************************************************

JI1201 TCCTCCTGAAGAATGGAGAAGCCCAATGTCCACATTCCAAGCTCTTTTTGCTATTCTTCT 2282
JI281  TCCTCCTGAAGAATGGAGAAGCCCAATGTCCACATTCCAAGCTCTTTTTGCTATTCTTCT 2303
JI2202 TCCTCCTGAAGAATGGAGAAGCCCAATGTCCACATTCCAAGCTCTTTTTGCTATTCTTCT 2302
Cameor TCCTCCTGAAGAATGGAGAAGCCCAATGTCCACATTCCAAGCTCTTTTTGCTATTCTTCT 2373
JI2822 TCCTCCTGAAGAATGGAGAAGCCCAATGTCCACATTCCAAGCTCTTTTTGCTATTCTTCT 2374
       ************************************************************

JI1201 TTCAAATGTATTACTGGTAACTTGGTTGATTTGTTTATATCCCGATTCTTTCCTCAGCTA 2342
JI281  TTCAAATGTATTACTGGTGACTTGGTTGATTTGTTTATATCCCGATTCTTTCCTCAGCTA 2363
JI2202 TTCAAATGTATTACTGGTAACTTGGTTGATTTGTTTATATCCCGATTCTTTCCTCAGCTA 2362
Cameor TTCAAATGTATTACTGGTAACTTGGTTGATTTGTTTATATCCCGATTCTTTCCTCAGCTA 2433
JI2822 TTCAAATGTATTACTGGTAACTTGGTTGATTTGTTTATATCCCGATTCTTTCCTCAGCTA 2400
       ****************** *****************************************

JI1201 GAGCTGGGTGGGTTTAGACGAAGCAAAATTCAGGTTGCAAGAATGACTATGAGAGGAATA 2402
JI281  GAGCTGGGTGGGTTTAGACGAAGCAAAATTCAGGTTGCAAGAATGACTATGAGAGGAATA 2423
JI2202 GAGC-------------------------------------------------------- 2366
Cameor GAGCTGGGTGGGTTTAGACGAAGCAAAATTCAGGTTGCAAGAATGACTATGAGAGGAATA 2493
JI2822 GAGC--------------------------------------------------------
       ****

JI1201 TGTAAAATTGCACAAAAACATATTACTTGTGCAATAAAGGAAATTTCATTTCATGAATTA 2462
JI281  TGTAAAATTGCACAAAAACATATTACTTGTGCAATAAAGGAAATTTCATTTCATGAATTA 2483
JI2202 ------------------------------------------------------------
Cameor TGTAAAATTGCACAAAAACATATTACTTGTGCAATAAAGGAAATTTCATTTCATGAATTA 2553
JI2822 ------------------------------------------------------------


JI1201 TGTAATTATGAAGTAAAGGAATTAACAGATTGGATTAAATCTCACTCAAAGATATGGCCT 2522
JI281  TGTAATTATGAAGTAAAGGAATTAACAGATTGGATTAAATCTCACTCAAAGATATGGCCT 2543
JI2202 ------------------------------------------------------------
Cameor TGTAATTATGAAGTAAAGGAATTAACAGATTGGATTAAATCTCACTCAAAGATATGGCCT 2613
JI2822 ------------------------------------------------------------
```

Start of transcription in *cv.* Cameor
SNP
Indel
Exon
START-STOP

S3b

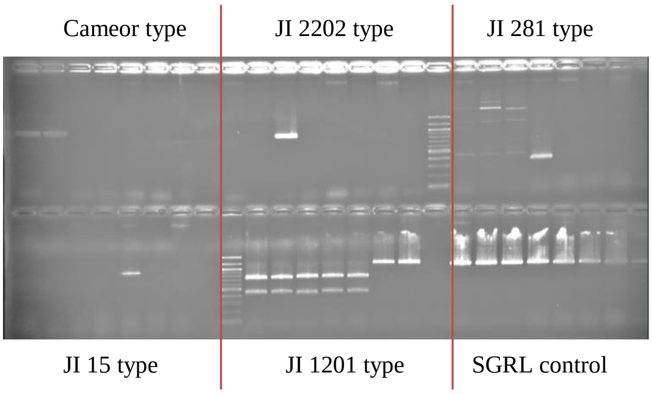

S3c

```
Cameor CATGCACTCTAGCATCATTTACAATTAATATTTTTTCAAGAATTGCTAAATTAAAGAATA 60
JI2822 TATGCACTCTAGTATCATTTACAATTAATATTTTTTCCAGAATTGCTAAATTAAAGAATA 60
        *********** ************************ **********************

Cameor TATTTTTATTAACGAATTAGAGAAATTTGTTAAAGGAGTATTCCCTCCATTTTTTATCAT 120
JI2822 TATTTTTATTAAAGAATTAGAGAAATTTGTTAAAGTAGGATTCCCTCTATTTTTTATTAT 120
       ************ ********************** ** ******** ********* **

Cameor AAATTATTTTGAAAAATAAAATTGTAAGTCAGTTTACACTACCAATAAATAAATAGTGTT 180
JI2822 AAGTCATTTTGAAAAATAAAATTATAAGTCAGTTTACACTACCAATAAATAAATAATGTT 180
       ** * ****************** ******************************* ****

Cameor ATTTTTT-TATTATACTTTTAAAAATTTATTATTATCTTTTATTTTAATTATATGACACA 239
JI2822 ATTTTTTCTATTATACTTTTAAAAATTTATTATTATCTCTTATTTTAATTATATAACACA 240
       ******* ****************************** *************** *****

Cameor TTAATAATATTATAAAATATTTATAATTTTTTATTTTCATACAATAATAATTATTATTTT 299
JI2822 TTAATAATATTATAAAATATCTATAATTTTTTATTTTCATACAATAATTATTATTTTTCT 300
       ******************** *************************** ****** ** *

Cameor TAATTACTTATGAAAAGTTCAAAAAGTTTTATAATAAATAGTATTTAATAGAG------- 352
JI2822 TAATTACTTATGAAAAGTTCAAAAAGTTTTCTAATAAAAAACGTTCAAAAGAATGTTGTA 360
       ****************************** ******* *   ** ** ***

Cameor -AAAGCAGGTCTGGGTGAGTTGATCCGAAACCGAACAGCTGATCCAAGTAAAGCCACCTC 411
JI2822 TAAAGCAGGTCTGGGTGAGTTGATCCGAAACCCAACCGCTGATCCAAGTAAAGCCACCTC 420
        ****************************** *** ************************

Cameor GTTACATTATCCATCCAAAAGTTATCGAAAATTTGAAGTATTTGATGACGTGGCACAGCA 471
JI2822 GTTACATTATCCATCCAAAAGTTATCGAAAATTTGAAGTATT-GATGACGTGGCACAGCA 479
       ****************************************** *****************

Cameor CATTCAATCCCATTCTCCATTACAATGTGTTTGTGTGTTTTAATTTTCCTATGATTAATG 531
JI2822 CATTCAATCCCATTCTCCATTACAATGTGTTTGTGTGTTTTAATTTTCCTATGATTAATG 539
       ************************************************************

Cameor TTAACTAATAAAATAAAGTAAAAAGAAAGAAAGAAAATGTGTGGAAGAAATGGATTGATA 591
JI2822 TTAACTAATAAAATAAAGTTAAAAGAAAGAAAGAAAATGTGTGGAAGAAATGGATTGATA 599
       ******************* ****************************************

Cameor GAGATTTTGGATTGTTGCGGTGTTTGAACAGAATCACACAGTCCACACGAACAAACCAAA 651
JI2822 GAGATTTTGGATTGTTGCGGTGTTTGAACAGAATCACACAGTCCACAAGAACAAACCAAA 659
       *********************************************** ************
```

```
Cameor CTGAGAACACTAATTTCCCACATCACTCCTTCTTTCTCTCTCTTTCTATCTCTAACTCTT 711
JI2822 CTGAGAACACTAATTTCCCACATCACTCTTTCTTTCTCTCTCTTTCTATCT-TAACTCTT 718
       **************************** ********************** ********

Cameor TTAACAGCAGAAGAACAAGGAAAGAAGATAATGGTGGTGACATGTGAAACATGAAGAGTA 771
JI2822 TTAACTACAGAAGAACAAGGAAAGAAGATAATGGTGGTGACATGTGAAACATGAAGAGGA 778
       *****  **************************************************** *

Cameor CTGTATTATTAT---GCATCTTCA 792
JI2822 CTGTATTATTATTATGCATCTTCA 802
       ************   *********
```

Start of transcription in *cv.* Cameor

Indel

Differences in predicted promoter motifs
