# Supplementary material for: SGRL can regulate chlorophyll metabolism and contributes to normal plant growth and development in Pisum sativum L
Source: Plant Mol Biol. 2015 Sep 7;89(6):539–58. doi: 10.1007/s11103-015-0372-4 (PMC4659853; doi:10.1007/s11103-015-0372-4)
Supplement: Supplementary file 8 — Sequence of pea SGRL (upper line) showing the predicted transmembrane domain (highlighted yellow); underneath are the protein regions which are common to all SGRL constructs that display activity in transient expression assays. The dashed line indicates the extent of deletions in constructs that did not abolish activity. The positions of early stop codons, reflecting that of a TILLING mutant (W197STOP) and two engineered mutants (S241STOP) and (M229STOP), are shown (red) (PDF 38 kb) [file 11103_2015_372_MOESM8_ESM.pdf]

**S8**

```
SGRL     MASLCHNAFSFSPTKLFPIMLKPSFRCSSITTNSTPSYNSIVFETVRLLGPPTKFEASKL 60
         MASLCHNAFSFSPTKLFPIMLKPSFRCSSITTNSTPSYNSIVFETVRLLGPPTKFEASKL
         ************************************************************

SGRL     KVVLLEDQINRYASIIPRTYILSHCDLTANLTLAVSNVIKLEQLRGWYQKDDVVAEWKKV 120
         KVVLLEDQINRYASIIPRTYILSHCDLTANLTLAVSNVIKLEQLRGWYQKDDVVAEWKKV
         ************************************************************

SGRL     KNEMCLHVHCFVSGPNSFLDLAAEFRYHIFSKEMPLVLKAIQCGDSELFHEHPELLDSIV 180
         KNEMCLHVHCFVSGPNSFLDLAAEFRYHIFSKEMPLVLKAIQCG----------------
         ********************************************

SGRL     RVYFHSSSKIYNRMECWGPLRDAMEGKRGDQLQGLINRDRPPEEWRSPMSTFQALFAILL 240
         ------------------------EGKRGDQLQGLINRDRPPEEWRSP----------
                                 ************************

SGRL     SNVLLVTWLICLYPDSFLS 259
         -------------------
```
