# Supplementary material for: SGRL can regulate chlorophyll metabolism and contributes to normal plant growth and development in Pisum sativum L
Source: Plant Mol Biol. 2015 Sep 7;89(6):539–58. doi: 10.1007/s11103-015-0372-4 (PMC4659853; doi:10.1007/s11103-015-0372-4)
Supplement: Supplementary file 11 — Supplementary material 11 (PDF 106 kb) [file 11103_2015_372_MOESM11_ESM.pdf]

Supplementary Table 1 List of primers used in this work

Purpose	Amplicon length (bps)	Primer names	Primer sequences
SGRL sequence identification	692	MK_C15f<br>MK_C15rev	CATAATGCCTTTTCATTTTCACC<br>TGTAGGTTCTTGGGATAATACTTGC
SGRL sequence identification	Nested primers for suppression PCR	SGRL-R1<br>SGRL-R2	AAAGATGGCTTTAACATGATAGGG<br>GGTGAAAATGAAAAGGCATTATG
SGRL sequence identification	Nested primers for suppression PCR	SGRL-F1<br>SGRL-F2	GCAAGTATTATCCCAAGAACCTACA<br>TCTATCCCACTGTGATTTAACAGC
SGRL sequence identification	Nested primers for suppression PCR	SGRL-F3<br>SGRL-F4	GTTTATGTTTGAGTCCGGAGG<br>ATTGCAACCTTATCGAAGAGTTG
SGRL JI 281 x JI 399 mapping	692, cut by SspI	MK_C15f<br>MK_C15rev	CATAATGCCTTTTCATTTTCACC<br>TGTAGGTTCTTGGGATAATACTTGC
SGRL Princess x JI 185 mapping	897<br><br>Sequencing primer	SGRL-F8<br>SGRL-R1<br>SGRL-R3	CGAACCTACTGTGTTTTTGGG<br>AAAGATGGCTTTAACATGATAGGG<br>ATCAACTCACCCAGACCTGC
SGRL diversity screen	1250	SGRL-F9<br>SGRL-R7	GATTTTGGATTGTTGCGGTG<br>TCATCCTTCTGGTACCACCC
SGRL diversity screen	1121	SGRL-F2<br>SGRL-R4	TCTATCCCACTGTGATTTAACAGC<br>GAATCTCCGCATTGAATTGC
SGRL diversity screen	1163	SGRL-F10<br>SGRL-R5	TTGCTTTGTTAGTGGTCCTAATT<br>GAATTTTGCTTCGTCTAAACCC
SGRL diversity screen	717	SGRL-F4<br>SGRL-R11	ATTGCAACCTTATCGAAGAGTTG<br>TCGGGATATAAACAAATCAACC
SGRL TILLING genotyping	736	SGRL-F4<br>SGRL-R5	ATTGCAACCTTATCGAAGAGTTG<br>GAATTTTGCTTCGTCTAAACCC
SGRL 'Cameor' specific	919	SGRL-F1<br>SGRL-diag3R	GCAAGTATTATCCCAAGAACCTACA<br>CTGAATCTTGTTTATTTGAGC
SGRL 'JI 2822' specific	929	SGRL-diag1F<br>SGRL-R4	CGTTTGATACTTCTTTTAAGTGGG<br>GAATCTCCGCATTGAATTGC
SGRL 'JI 2202' specific	881	SGRL-diag5F<br>SGRL-R4	CTTAGGTTGTTCATTAGTATATCC<br>GAATCTCCGCATTGAATTGC
SGRL 'JI 281' specific	351	SGRL-diag6F<br>SGRL-R7	CCTACATTCTATCCCACTCC<br>TCATCCTTCTGGTACCACCC
SGRL 'JI 1201' specific	1200, cut by BamHI to 750 and 350	SGRL-F10<br>SGRL-R5	TTGCTTTGTTAGTGGTCCTAATT<br>GAATTTTGCTTCGTCTAAACCC
SGRL 'control' for allele specific PCR	1121	SGRL-F2<br>SGRL-R4	TCTATCCCACTGTGATTTAACAGC<br>GAATCTCCGCATTGAATTGC
SGRL 5'UTR limit identification	Gene specific primers used	SGRL-R4<br>SGRL-R7	GAATCTCCGCATTGAATTGC<br>TCATCCTTCTGGTACCACCC
SGRL 3'UTR limit identification	Gene specific primers used	SGRL-F10<br>SGRL-F6	TTGCTTTGTTAGTGGTCCTAATT<br>TCCTGAAGAATGGAGAAGCC

SGRL constructs	841/838 (HIS)	attB1-SGRL-F<br>attB2-SGRL-R<br>attB2-SGRL-R-HIS	GGGGACAAGTTTGTAAAAAAAGCAGGCTCAATGGCATCATTATGTCATA<br>GGGGACCACTTTGTACAAGAAAGCTGGGTTCTAGCTGAGGAAAGAATCGG<br>GGGGACCACTTTGTACAAGAAAGCTGGGTAGCTGAGGAAAGAATCGGGAT
SGR constructs	847/844 (HIS)	attB1-SGR-F<br>attB2-SGR-R<br>attB2-SGR-R-HIS	GGGGACAAGTTTGTACAAAAAAGCAGGCTACATGGATACTCTAACGAGTG<br>GGGGACCACTTTGTACAAGAAAGCTGGGTTTTACAAGTTACCATGTTGGG<br>GGGGACCACTTTGTACAAGAAAGCTGGGTACAAGTTACCATGTTGGGGAT
PaO constructs	1660/1657 (HIS)	attB1-PaO-F<br>attB2-PaO-R<br>attB2-PaO-R-HIS	GGGGACAAGTTTGTACAAAAAAGCAGGCTTGATGGCACTTCCACACACAC<br>GGGGACCACTTTGTACAAGAAAGCTGGGTTTCAATCGATTTCGGCATGTA<br>GGGGACCACTTTGTACAAGAAAGCTGGGTAATCGATTTCGGCATGTAACT
SGRL3421 HIS tagged construct	649 (HIS)	attB1-SGRL-F<br>attB2-3421-HIS	GGGGACAAGTTTGTAAAAAAAGCAGGCTCAATGGCATCATTATGTCATA<br>GGGGACCAGTTTGTACAAGAAAGCTGGGTAACATTCCATTCTGTTGTAT
SGRL (S241^{STOP}) constructs	784/781 (HIS)	attB1-SGRL-F<br>attB2-S241^{STOP}-R<br>attB2-S241-HIS	GGGGACAAGTTTGTAAAAAAAGCAGGCTCAATGGCATCATTATGTCATA<br>GGGGACCACTTTGTACAAGAAAGCTGGGTTTCAAAGAAGAATAGCAAAAA<br>GGGGACCACTTTGTACAAGAAAGCTGGGTTAAGAAGAATAGCAAAAAGAG
SGRL (M229^{STOP}) constructs	748/745 (HIS)	attB1-SGRL-F<br>attB2-M229^{STOP}-R<br>attB2-M229-R-HIS	GGGGACAAGTTTGTAAAAAAAGCAGGCTCAATGGCATCATTATGTCATA<br>GGGGACCACTTTGTACAAGAAAGCTGGGTACTATGGGCTTCTCCATTCTT<br>GGGGACCACTTTGTACAAGAAAGCTGGGTATGGGCTTCTCCATTCTTCAG

SGRL qPCR primers	104	SGRL-F<br>SGRL-R	GCAATGGAGGGAAAACGAGGC<br>GCAAAAAGAGCTTGGAATGTGG
SGR qPCR primers	139	QSGR-2F<br>QSGR-2rev	CGCAATGGAAGAAGGTGAAG<br>AAAGCCTTCAACACCACAGG
Actin qPCR reference gene	104	PsActF<br>PsActR	CACAATTGGCGCTGAAAGAT<br>TAATTGAGTTAAATGTCGTCTCATGGA

pDONR vector sequence checks	Variable	pDONR-Fs<br>pDONR-R	GTTAACGCTAGCATGGATCTC<br>GTAACATCAGAGATTTTGAGACAC
DEST 1/3 vector sequence checks	Variable	M13-F<br>M13-R	GTAAAACGACGGCCAGTG<br>GGAAACAGCTATGACCATG
DEST 1/3 vector sequence checks	Variable	attB1-Fs<br>attB2-Rs	ACAAGTTTGTACAAAAAAGCAGGC<br>ACCACTTTGTACAAGAAAGCTGGG

Suppression PCR adaptor pair	use with BamHI, BclI, BglII, BstYI	APX1 top<br>APX1 lower	GATCACCTGCCCAA<br>CGTCTGATGCTGACTACGCTTGCATCGTCGCTCCGGGCAGGT
Suppression PCR adaptor pair	use with ClaI, MspI, TaqI	TPX1 top<br>TPX1 lower	CGTCGTGCGTGCAA<br>CGTCTGATGCTGACTACGCTTGCATCGTCGCTCCGCACGCACGA
Suppression PCR adaptor pair	use with DraI, HpaI, EcoRV, NaeI, ScaI, PvuII, SspI, StuI	APX1Blunt top<br>APX1Blunt lower	ACCTGCCCAA<br>CGTCTGATGCTGACTACGCTTGCATCGTCGCTCCGGGCAGGT
Suppression PCR primers	Variable	APX1A<br>APX1B	CGTCTGATGCTGACTACGCT<br>ACTACGCTTGCATCGTCGCT

cDNA synthesis primer<br>Poly-A adaptor primer		A236<br>A237	GACTCGAGAATTGGATCCTTTTTTTTTTTTTTTTTT<br>GACTCGAGAATTGGATCC
